# A Comprehensive Analysis of Methods for Improving and Estimating Energy Efficiency of Passive and Active Fiber-to-the-Home Optical Access Networks

**DOI:** 10.3390/s25196012

**Published:** 2025-09-30

**Authors:** Josip Lorincz, Edin Čusto, Dinko Begušić

**Affiliations:** 1 Faculty of Electrical Engineering, Mechanical Engineering and Naval Architecture (FESB), University of Split, 21000 Split, Croatia; 2 Croatian Academy of Engineering, 10000 Zagreb, Croatia

**Keywords:** power, energy efficiency, PON, AON, GPON, FTTH, point-to-point, modeling, optical, access, networks, OLT, ONU, distribution, power-saving techniques, resource management

## Abstract

With the growing global deployment of Fiber-to-the-Home (FTTH) networks driven by the demand for ensuring high-capacity broadband services, mobile network operators (MNOs) face challenges of excessive energy consumption (EC) of wired optical access networks (OANs). This paper presents a comprehensive review of methods aimed at improving the energy efficiency (EE) of wired access passive optical networks (PONs) and active optical networks (AONs). The most important energy management and power-saving methods for Optical Line Terminals (OLTs) and Optical Network Units (ONUs), as key OAN components, are overviewed in the paper. Special attention in the paper is further given to analyzing the impact of a constant increase in the number of subscribers and average data rate per subscriber on global instantaneous power and annual energy consumption trends of FTTH Gigabit PONs (GPONs) and FTTH point-to-point (P-t-P) networks. The analysis combines the real ONU/OLT device-level power profiles and the number of installed OLT and ONU devices with data traffic and subscriber growth projections for the period 2025–2035. A comparative EE analysis is performed for different MNO FTTH OAN architectures and technologies, point-of-presence (PoP) subscriber capacities, and GPON-to-P-t-P subscriber distribution ratios. The findings indicate that different FTTH PON and AON architectures, FTTH technologies, and PON-to-AON subscriber distributions can yield significantly different EE gains in the future. This review paper can serve as a decision-making guide for MNOs in balancing performance and sustainability goals, and as a reference for researchers, engineers, and policymakers engaged in designing next-generation wired optical access networks with minimized environmental impact.

## 1. Introduction

The implementation of fiber-optical technology is seen as a great contributor to improving the telecommunication network energy efficiency (EE). For example, the implementation of fiber-optical technology in fiber–wireless networks contributes to the improvement of the EE of wireless radio access networks [1]. However, different parts of a wired optical network, such as metro, core/transport, or access networks, have different demands in terms of performance and EE [2]. The exponential growth of Internet traffic and the proliferation of data-intensive applications have placed unprecedented demands on the capacity, performance, and EE of wired optical access networks.

In order to satisfy such implementation demands, Fiber-to-the-Home (FTTH) networks, also known as Fiber-to-the-Premises networks, are seen as the ultimate technology in the wired access network segment, for which it is expected to eventually be implemented as a full substitution for copper-based wired access technologies. The FTTH network is an optical-fiber-based telecommunication technology that is utilized for delivering high-data-rate broadband Internet from Mobile Network Operator (MNO) premises directly to individual subscribers (users), which can be businesses, public users, private users, etc.

A side effect of this constantly increasing global penetration of FTTH networks and, consequently, the increase in the number of FTTH subscribers expecting seamless, high-data-rate connectivity is that MNOs face challenges related to appropriately scaling the capacity of FTTH network infrastructure while minimizing network energy consumption (EC). Although FTTH passive optical networks (PONs) and active optical networks (AONs) have emerged worldwide as leading solutions for meeting escalating bandwidth requirements in wired access and transport parts of mobile network operator (MNO) networks, the challenge of optimizing the EC of FTTH networks has become critically important. This importance stimulates intensive research, standardization efforts, and technological innovation in this area.

The FTTH PONs and AONs have different impacts on MNO EC and divergent contributions to the improvement of network EE. Generally, FTTH PONs exhibit lower total network energy consumption compared to their active counterparts. This is primarily because FTTH PONs typically employ passive optical devices such as optical splitters in the Optical Distribution Network (ODN), thereby reducing network power needs by eliminating active components that consume energy at intermediate points. By contrast, FTTH AONs introduce active switching or routing elements within the ODN, enabling greater capacity and flexibility in traffic management, which consequently contributes to increasing network power consumption. However, both FTTH PON and AON architectures also present substantial opportunities for EC reduction during no- or low-traffic periods, which can be realized through the implementation of sophisticated network device resource control and management strategies. Consequently, fostering better optical network EE demands a broad approach that integrates architectural design and dynamic network resource management mechanisms.

The main active elements that contribute to the overall power consumption in AON or PON FTTH networks are Optical Line Terminals (OLTs) located at the MNO side and Optical Network Units (ONUs) installed at the subscriber (user) side. Nevertheless, advanced energy-saving strategies have been developed to effectively reduce the instantaneous power consumption of OLTs and ONUs, especially during low- and off-peak traffic periods. These strategies can range from employing adaptive link rate (ALR), selective component deactivation, and sleep/doze/deep/watchful modes of operation to adjusting the optical split ratio and dynamic bandwidth/wavelength allocation [3,4].

Since objectives for simultaneously ensuring higher FTTH network transmission data rates and network sustainability continuously evolve on a global level, balancing between demands for high FTTH network capacities and low network EC remains a key objective for the realization of wired optical access networks worldwide. In alignment with these needs, the different standardization organizations have defined different metrics for expressing optical network EE and methods to optimize OLTs’ and ONUs’ power usage.

Despite the availability of various standardized energy-saving features and guidelines for improving optical network EE, the EE of real FTTH PONs and AONs depends on numerous factors. Some of them include specifics of network type and architecture, traffic load patterns, and techniques implemented by OLTs and ONUs for improving EE. These factors can significantly influence the optical network’s actual power consumption. Therefore, this review paper provides a comprehensive analysis of FTTH PON and AON EC and overviews methods for improving the EE of ONUs and OLTs, as the main elements of FTTH PONs and AONs. By examining the specific EC optimization features of PONs’ and AONs’ principal components, ranging from transmit/receive functionality of OLTs located in MNO point-of-presence (PoP) facilities to diverse functionalities of ONUs on the subscriber side, this paper offers structured insights into the primary factors that shape EC in optical access networks and enable its optimization. Additionally, this work presents a comprehensive analysis of the impact of subscriber and data rate increases on FTTH PONs’ and AONs’ global EC trends for the period 2025–2035. The estimation is performed based on real ONU and OLT power consumption profiles for the GPON as a characteristic representative of FTTH PONs, and point-to-point (P-t-P) FTTH networks as a typical representative of AONs.

The rest of the paper is organized as follows: Section 2 provides an overview of previous research on improving EE in ODNs, highlighting key technological, architectural, and methodological advancements dedicated to improving optical network EE. In Section 3, a comparison of the EC profiles for FTTH PON and AON architectures is presented, illustrating how passive signal splitting versus active switching influences the overall power consumption in PONs and AONs, respectively. Operating modes of ONU devices for reducing network energy consumption are analyzed in Section 4. Section 5 presents resource management techniques for optimizing the energy consumption of ONU and OLT devices. Future trends in the global EC of FTTH GPONs and FTTH P-t-P networks, impacted by the constantly increasing number of subscribers and data rates, have been modeled and are discussed in Section 6. Section 7 concludes the paper by synthesizing the main findings and future research directions.

## 2. Related Work

FTTH networks are significant contributors to the overall EC of MNOs. To enhance the EE of OLT and ONU devices, which are the primary energy consumers in OANs, various methods and recommendations have been introduced in both standards and the scientific literature.

### 2.1. Main Standards Related to Improving Optical Network EE

Among the most important standards related to improving ONU and OLT EE is Recommendation ITU-T G.984.2. Recommendation ITU-T G.984.2 introduces a power-leveling mechanism for ONU transmitters, which dynamically adjusts the optical signal strength. This approach effectively reduces the receiver dynamic range at the OLT side, enhancing EE and prolonging the transmitter lifespan. Its strength lies in its simplicity and direct impact on transmission power. However, it is limited in scope, as it does not address idle-state power management [5].

To achieve flexible transitions between low-power and active states, Recommendation ITU-T G.987.3 defines detailed energy management modes for ONUs. These modes include doze, cyclic sleep, and watchful sleep operating states, which are coordinated via ONU management and control interface (OMCI) signaling from the OLT to optimize network EE during periods of low traffic [6]. This specification’s strength is its comprehensive approach to reducing power during low-traffic periods. A limitation is the potential complexity in accurately synchronizing transitions without service disruption.

Additionally, Recommendation ITU-T G.9802 outlines dynamic wavelength tuning and channel reassignment strategies in multi-wavelength PON (MW-PON) systems, where ONU devices can be shifted to operate with fewer active channels during off-peak data traffic hours. The proposed concept, supported by Dynamic Wavelength and Bandwidth Assignment (DWBA) algorithms, ensures the efficient use of ONU device resources and power savings [7]. Recommendation relevance to improving EE is substantial, although its implementation requires advanced wavelength management and hardware support.

Supplement 45 to ITU-T G-Series Recommendations provides a comprehensive taxonomy and comparative analysis of energy-saving techniques for ONU and OLT devices, which include cyclic sleep, power shedding, and line-rate adaptation [8]. It also proposes signaling mechanisms to manage power-saving transitions effectively, without compromising service quality. The Recommendations’ strength lies in offering a comparative evaluation framework and standardized signaling methods, although its broad scope may require tailoring to specific network architectures.

Furthermore, Recommendation ITU-T L.1310 establishes standardized methodologies for evaluating EE in GPON and Gigabit Ethernet Passive Optical Network (GEPON) systems. The Recommendation introduces an Energy Efficiency Rating (EER) metric that defines data throughput per watt of power consumption under varying traffic loads as a relevant metric, thereby enabling comparative assessment of ONU and OLT equipment performance in controlled test environments [9]. This standard is crucial for EE benchmarking, but may not reflect real-time dynamic behaviors in live networks.

The IEEE 802.3av standard enhances EE in 10 Gbit/s Ethernet PON (EPON) systems through several mechanisms [10]. A key mechanism is a dual-rate operation, which allows the OLT to simultaneously handle 1 Gbit/s and 10 Gbit/s upstream signals using shared optical infrastructure. This approach reduces the need for additional hardware and enables more flexible deployment strategies, thereby minimizing network power consumption associated with redundant network components. Additionally, the standard mandates implementation of the Forward Error Correction (FEC) process, which increases the optical budget and reduces retransmissions, thus leading to lower network energy expenditure per transmitted bit of OLT and ONU devices. The implementation of dynamic bandwidth allocation (DBA) standardized in the IEEE 802.3av standard further optimizes the importance of upstream resource allocation, which results in reducing ONU idle periods and enables ONUs to enter low-power states during periods of data traffic inactivity. Also, discovery window protocols defined in IEEE 802.3av enable minimizing continuous signaling in optical networks, contributing to overall power savings in both OLT and ONU components [10]. These mechanisms reduce hardware redundancy and idle signaling, contributing significantly to OLT and ONU EE. Its comprehensive coverage is a major strength, though implementation complexity and backward compatibility issues are potential challenges.

The standard IEEE 1904.1 introduces a detailed framework for energy-saving operation in EPONs by implementing sleep control mechanisms for ONUs and OLTs [11]. The standard IEEE 1904.1 defines multiple low-power modes, such as transmit (Tx) sleep mode and transmit/receive (Tx/Rx) sleep mode, which selectively disable transmission and reception of ONU and OLT circuitry during low-traffic periods. It specifies power-on and power-off delays to coordinate smooth transitions between active and idle ONU and OLT states without compromising network stability. Furthermore, mechanisms like synchronized wake-up and early wake-up are included to support time-sensitive services (such as Voice over Internet Protocol (VoIP) or Internet Protocol Television (IPTV)), by preemptively reactivating ONUs when traffic demands increase. Additionally, the introduction of control signaling through Type-Length-Value (TLV) messages (e.g., Sleep Allow, Wake-Up) ensures efficient ONU state coordination, enabling substantial reductions in EC while preserving Quality of Service (QoS) in the network [11]. This standard’s strong alignment with QoS preservation while improving EE is a key advantage, though the added signaling overhead may affect scalability.

ETSI complements optical network standardization efforts by developing measurement methodologies and performance benchmarks for uniformly evaluating versatile optical network equipment EE [12]. These frameworks are valuable for guiding vendor design and deployment strategies, though adapting them to evolving technology standards may require frequent updates.

### 2.2. Literature Review on Improving ONU and OLT Device EE

An important mechanism for improving OAN EE is a mechanism related to putting parts or complete optical network elements in low-power (also known as sleep) mode during periods of low traffic activity. Sleep mechanisms, although effective in reducing the EC of network elements, can increase failures in optical networks. As shown in [13], the trade-off between improving network EE and increasing failures can be acceptable in residential networks with up to 22% Mean Time Between Failures (MTBF) reduction, but in business networks, the margin is limited to 5% due to higher costs of equipment failures. This highlights a key limitation of improving network EE in operational environments, which is the network’s sensitivity to failures.

In Ref. [14], based on the standard ITU-T Y.3022, a formal model for evaluating energy savings in Time Division Multiplexing (TDM) PONs is analyzed. Analyses show that operating modes of ONU and OLT devices, like “dose” and “cyclic sleep,” can reduce energy use by 30–50% depending on traffic load. This confirms the practical relevance of these operating modes and also suggests that EE performance varies with traffic conditions.

A broader analysis in [15] extends to the implementation of Wavelength Division Multiplexing (WDM) and Time and Wavelength Division Multiplexed (TWDM) in PONs and AONs, and Orthogonal Frequency Division Multiple Access (OFDMA) in PONs. Analyses highlight techniques such as cyclic sleep, bit interleaving (Bi) PON, adaptive low-power transceivers, and band filtering, which are implemented in combination or as standalones in PONs. Implementation of these methods enables measurable EC savings, particularly through optical network devices with adaptable laser power and frequency control, offering a foundation for energy-efficient optical network design.

The authors of [16] present a unified approach to energy optimization of ONU and OLT devices in PONs, focusing on sleep/doze modes, adaptive transmission rates, and traffic aggregation via DBA protocols. It is also shown in [16] that techniques such as cyclic ONU wake-up and partial link deactivation enable improved optical network EE, though precise timing control remains challenging.

In Ref. [17], sleep mode optimization and split ratio adjustment are analyzed for a GPON, 10 Gigabit PON (XG-PON), and TWDM-PON. Algorithms like Sleep Mode Aware (SMA) and Hybrid Sleep Mode Aware (HSMA) are shown to reduce EC in optical networks by up to 60%, highlighting the importance of implementing a low-power operating mode in the resource management of OLT and ONU devices in OANs. The analyses presented in [17] emphasize the potential of intelligent sleep mode management, which requires sophisticated traffic prediction models.

For EPONs, Reference [18] proposes an ONU-driven sleep mode optimization where ONUs enter doze sleep modes autonomously using the Multi-Point Control Protocol (MPCP)-2 schedule, which does not require extra OLT signaling. This simplifies ONU coordination and enables 52.5–56% energy savings for ONU devices with minimal software changes. This is particularly relevant due to its ease of deployment, though its applicability may be limited to certain protocol versions.

In Ref. [19], similar power-saving techniques for ONU and OLT devices, including power shedding, deep sleep, and cyclic sleep within the XG-PON, are explored with traffic grouping and efficient DBA use. These methods offer good energy savings but may require service delay tolerance. Additionally, Ref. [20] introduces the “Green Meter” methodology, showcasing the role of network function virtualization (NFV) in enhancing optical network EE. The NFV strength is in centralizing resource control, but integration complexity can be a barrier.

In Ref. [21], traffic data from the FTTH network were used for analyses of AON and PON energy consumption. The results of analyses showed that, under specific conditions during low-traffic periods, the implementation of the dynamic switching (active/idle/off) mode of ONU and OLT operation can lead to device energy savings of up to 67%. Moreover, by extending these access network results to the end-to-end network perspective, the overall potential for energy savings of the entire optical FFTH network can reach up to 31% through the use of energy-aware equipment.

According to [22], FTTH-GPONs have the highest EE, with 95% of energy use on the user side (ONUs/gateway/router), and stand out as the most energy-efficient broadband access technology.

The reviewed standards and research offer a range of mechanisms with proven or potential benefits for improving the EE of optical access networks. While many techniques show strong energy-saving capabilities, their effectiveness is often influenced by network architecture, traffic patterns, and service requirements. Building on theoretical and empirical findings presented in previously published works and standards, this work overviews methods and approaches for improving the EE of active and passive FTTH networks. The analysis considers techniques and methods for optimizing the EE of OLT and ONU devices as the main active elements of FTTH networks.

## 3. Comparison of PON and AON Power Consumption

This section presents a comparative analysis of FTTH PONs and AONs in terms of network architecture, emphasizing their EE characteristics and performance. The section explores how different passive and active FTTH network architectures influence network EC and contribute to the overall network EE. Additionally, in this section, a comparison between PONs and AONs in terms of energy consumption is analyzed.

### 3.1. Architecture of PONs and AONs

**Figure 1:**
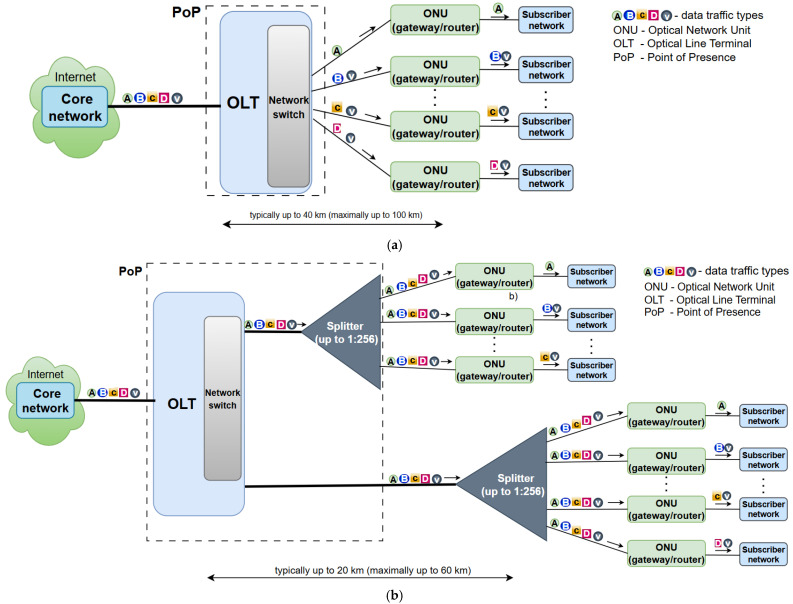
FTTH network: (**a**) AON architecture [23], (**b**) PON architecture [24].

Figure 1 presents the differences between the FTTH network architectures of AONs and PONs. The architecture of PONs is based on utilizing a passive (lacking a need for power supply) ODN that distributes optical signals between the OLT and ONUs. The active components in PONs are limited to the OLTs (network switches) typically located in MNOs’ PoP and ONUs, which are connected over passive optical splitters (Figure 1b). The passive optical splitters split the optical signal with optical splitting ratios that are, in practice, typically equal to 1:8/16/32/64/128/256 splitting ratio combinations. Due to its passive nature, passive optical splitters(s), on the one hand, eliminate the need for a power supply, and this contributes to the reduction in optical network power consumption. On the other hand, splitting the optical signal into multiple equal optical signals proportionally reduces the optical signal strength of each split optical signal. This splitting of signals consequently has an impact on reducing the split optical signal transmission range (Figure 1b). The typical optical signal transmission range between the OLT and ONU in a Gigabit PON (GPON) is 20 km, and the maximum transmission range, according to ITU-T G.984.2, with implementation of advanced optics and under optimal conditions, can be up to 60 km [5]. ONUs located on the subscriber side connect the subscriber’s network with the optical access network (Figure 1). In practice, the ONU can be implemented as a separate device, or it can integrate some or all of the functions of the router, gateway, and wireless local area network (WLAN) access point. In this work, the ONU is assumed to be a device that integrates ONU functions with router, gateway, and WLAN functionalities in a single device.

Unlike PONs, AONs, presented in Figure 1a, depend solely on active (power-consuming) network nodes such as OLTs (network switches) and ONUs, which, for optical signal distribution in FTTH networks, ensure one-to-one mapping between the optical port of the OLT (network switch) and the ONU device on the subscriber side [3,4]. The PoP hosting OLT device ensures the protected placement and power supply of the OLT device and also sometimes contains allocated PoP ancillary equipment that can include air conditioning and a monitoring system. OLT devices in practice incorporate a large number of optical ports and a network switching function, which is performed in the electrical domain after opto/electric conversion of signals and their transmission in optical form after electro/optic conversion. Since an AON does not exploit passive optical splitters and instead uses a direct optical connection between the OLT (network switch) and ONU (Figure 1a), an AON allows data transmission over greater distances compared with a PON. These distances are typically up to 20 km as a common standard and can go up to 40 km with implementation of amplified systems or lower-loss fiber, while, in some specialized implementations, they can range up to 80–100 km (Figure 1a). However, AONs are more energy demanding than PONs, since a larger number of optical ports and consequently active network equipment (OLTs) need to be engaged to ensure P-t-P connection based on one-to-one mapping between each OLT (network switch) optical port and each ONU device on the subscriber side (Figure 1a).

### 3.2. EC of PONs

Within PONs, ONU devices are responsible for over 65% of total EC [3]. The largest presented power consumers in ONU devices are the Clock and Data Recovery (CDR) and Serializer/Deserializer (SERDES) components. These two components together consume more than 80% of the total instantaneous power of the front part of the ONU receiver [9,23]. Table 1 presents the values of measured and estimated instantaneous power consumption of various ONU components in different PON types (which include EPON, GPON, 10-Gigabit EPON (10G-EPON), and XG-PON) [3].

The data presented in Table 1 demonstrates that although ONU devices for newer PON technologies offer increased performance, they also have higher energy demands. According to [25], 10G-EPON technology includes components such as a burst-mode receiver (which includes the burst-mode transimpedance amplifier (B-TIA), burst-mode limiting amplifier (B-LA), and burst-mode clock and data recovery (B-CDR)). Burst mode refers to receivers that must process variable input optical signals at a high data rate of 10 Gbit/s, while maintaining minimal power consumption. The document [25] specifies requirements for a fast response time to ensure high efficiency of signal processing in PoPs (OLTs). Receiver circuits such as B-TIA, B-LA, and B-CDR are optimized to reduce power consumption. B-TIA uses a dynamic gain adjustment mechanism to prevent excessive power consumption at strong signals [25]. PONs are characterized by higher EE due to passive elements in the distribution infrastructure, eliminating the need for active devices such as aggregation switches. However, advanced PON systems, such as 10G-PON and Next-Generation (NG)-PON2, are characterized by an increase in energy consumption due to more complex protocols that govern optical signal transmission and the need to ensure higher data rates [6].

A comparison of typical performance and power consumption parameters for the most common practically implemented PON types, which include the GPON, 1G-EPON, 10G-EPON, and XG-PON (10G-PON), is summarized in Table 2 [25]. While all PON technologies are based on a point-to-multipoint (P2MP) architecture, according to Table 2, the PON EE significantly varies depending on bandwidth and user (subscriber) density. PON technologies such as GPONs and EPONs stand out as the most energy-efficient options due to their passive infrastructure and support for power-saving modes like sleep and power-off states [3]. The GPON and 1G-EPON offer better EE at moderate user loads, while 10G-EPON demonstrates outstanding performance in high-density scenarios with many users [26]. Next-generation solutions such as 10G-EPON and XG-PON further enhance EE through advanced features like dynamic bandwidth allocation, high transmission data rates, and integrated power management mechanisms (Table 2) [6]. Although the GPON is less efficient in small-scale networks, it becomes significantly more energy-efficient beyond approximately 1500 subscribers (users), as the fixed EC of central components (e.g., OLT) is distributed over a larger user base, reducing the per-user energy cost [26].

XG-PON adds to this EE improvement option of Watchful Sleep Mode of operation for ONU devices, which can achieve up to 65% power savings while maintaining acceptable delay and throughput trade-offs [31]. As a result, GPON, in terms of achieving energy-efficient network implementations, remains a viable solution for densely populated FTTH deployments, where infrastructure sharing plays a critical role in optimizing energy consumption. According to [32], PONs benefit from their passive and shared architecture, resulting in lower per-user (subscriber) EC (approximately 6–7 W) and improved efficiency at medium data rates, achieving 0.03 µJ/bit at 100 Mb/s. For this reason, the GPON was selected as a representative of FTTH PONs for the EC modeling and analysis of future global FTTH PONs presented in the last part of this paper.

### 3.3. EC of AONs

AONs consume more energy compared to PONs serving the same number of subscribers due to the need for a larger number of active components, such as aggregation switches and corresponding optical ports. These networks offer high data transfer rates, while network EE can be optimized using advanced optical device scheduling techniques, such as adaptive control of data transfer rates and switching off inactive links [6]. However, AONs can face limited possibilities for reducing network EC due to their need to have constantly powered-on active components such as network switches [16]. A comparison of performance parameters and power consumption of P-t-P 1G Ethernet, P-t-P 10 Gbit/s Ethernet, and P-t-P 100/200/400 Gbit/s Metro Aggregation Ethernet technologies is presented in Table 3.

Table 3 indicates that P-t-P 10G/100/200/400 Gbit/s optical network architectures, although offering potential for achieving high data rates and data transmission efficiency, have sometimes limited practical deployment capabilities due to high energy demands in the case of low user densities and uplink capacity constraints of the MNO central office (CO) and PoPs. For this reason, a P-t-P 1 Gbit/s AON was selected in the last part of this paper as a representative of FTTH AONs for the modeling and analysis of future global FTTH AONs’ EC.

**Figure 2:**
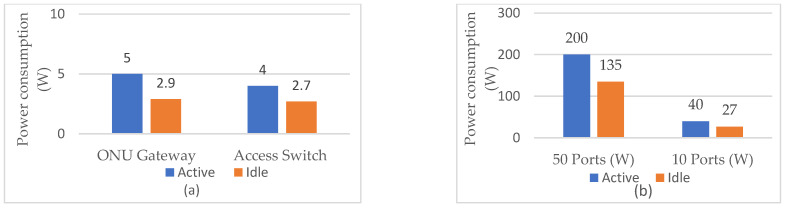
(**a**) Power consumption of ONU (gateway) and access switch port in active and idle operating state [21]; (**b**) power consumption of a network switch (SW) having different numbers of active and idle ports [21].

According to [21], AONs exhibit significantly higher energy consumption compared with PONs, primarily due to the continuous power requirements of active intermediate nodes located in PoPs (Figure 1). Figure 2 presents the measured power consumption for the ONU (router/gateway) and network switch in active and idle operating states and for network switches having different numbers of active and idle ports [21]. Network switches, which are essential components of AONs, consume an average of 4 W per port in the active state and 2.7 W in the idle state (Figure 2a). In comparison, a single ONU device consumes on average 5 W when in active mode and 2.9 W in idle operating mode. Figure 2 also shows that the EC of OLTs with network (Ethernet) switches and ONUs (router/gateway) can have strong variations and depend on the mode of operation and the number of ports that are in the active or idle state. When it comes to large networks, optimizing the number of active switch ports and using different operating modes of switch ports can further reduce the total OLT energy consumption.

Furthermore, studies of AONs’ power consumption indicate that EC can be reduced by up to 67% through the application of the idle mode of operation for optical network devices (router gateway, access switches) [32]. According to the example presented in [32], a simple P-t-P network topology in which each user is connected through an Ethernet switch, with a total power consumption of 466 W and a capacity of 72 Gbit/s, allows simultaneous connection for up to 72 subscribers (users) at a data rate of 1 Gbit/s per subscriber (user) per switch port. While this configuration is slightly less energy-efficient than AON configurations operating at higher transmission data rates (10/100/200/400 Gbit/s), it is shown in [32] that a 1 Gbit/s AON configuration achieves EC approximately equal to 0.008 µJ/bit, while the customer-side ONU in P-t-P networks consumes on average 4 W, and the energy per bit improves with increasing data rate, decreasing from 2 µJ/bit at a 1 Mb/s data rate to approximately 0.005 µJ/bit at 1 Gbit/s. According to [38], these findings are further supported by the observation that P-t-P architectures typically require more extensive infrastructure and lead to higher power consumption, often exceeding 10 W per subscriber (user).

### 3.4. Differences Between the Power Consumption of PONs and AONs

In Ref. [39], a comparative overview of power consumption between the main optical access network types (PON and AON) is presented based on simulation results under varied network conditions. The data is presented for the two network operating methodological frameworks: the power-ignoring method, which assumes that network components remain fully powered regardless of data transmission activity, and the power-saving method, which implements a basic sleep mode mechanism in OLT and ONU devices to reduce energy consumption during idle traffic periods. For the power-ignoring method, both the PON and AON maintain full operating status continuously, leading to energy losses when no data is transmitted. In the analyses, baseline power demands of each architecture across three scenarios are highlighted. The analyzed scenarios include the ideal operating case (which represents minimum power consumption), worst operating case (which represents maximum power consumption with maximum losses and cooling overhead), and optimized operating case (which represents improved EE through parameter tuning). The power-saving method introduces a protocol enabling ONUs to enter a temporary sleep mode when not in use, effectively powering down key components, such as the photodiode, transimpedance amplifier, and limiting amplifier.

The simulation results related to the obtained total power consumption of the access network path from the OLT to the ONU (Figure 1) for network architectures without and with implemented power-saving methods are presented in Table 4 and Table 5, respectively [39]. According to the presented results, the implementation of the power-saving method reduces EC substantially in both network types (PON and AON). However, the PON, due to its passive nature and lower amount of optical network infrastructure, consistently demonstrates superior power consumption reductions across all operating conditions. AON architecture relies on an active network switch in the PoP, which consumes 64 W serving 32 subscribers (users). This significantly increases the overall network power consumption of the access network path. However, continuous operation of the equipment in the power-ignoring operating mode limits the potential for energy savings, since sleep mode is not applied (Table 4). For the implementation of a power-saving method in the PON, there is no active equipment in the access network path from the OLT to the ONU, and sleep mode applies to both the OLTs and ONUs, enabling the highest power consumption reductions (Table 5). Although the AON achieves reduced power consumption when the power-saving method is implemented, its instantaneous power consumption is higher than that of the PON (Table 5) due to a larger amount of OLT equipment needed to serve the same number of subscribers compared to the PON configuration. Therefore, the overall potential for energy savings in AONs is lower than that in PONs.

Table 6 presents a comparative analysis of OLT and ONU device power consumption for AON and PON architectures in both active and sleep modes [40]. Table 6 shows that AON devices can have somewhat lower per-device power consumption in comparison with network devices in different PON types, which is in contrast with general expectations related to the PON’s lower overall power consumption due to its passive design. Among PON variants, the TWDM PON achieves the lowest OLT power per user due to centralized wavelength management, while the WDM PON allows more effective application of sleep modes at the ONU side. Despite favorable device-level metrics in the AON (Table 6), these values exclude the additional power demands of intermediate active components such as Ethernet switches. Therefore, an assessment of the overall optical network EE needs to be performed in the context of the complete network architecture.

## 4. Operating Modes of ONU Devices for Reducing Network Energy Consumption

The next section analyzes different operating modes of ONU devices for improving optical network EE. The analyzed operating modes of ONU devices and their corresponding characteristics are presented in Table 7. The analyzed operating modes include sleep/deep sleep, cyclic sleep, doze, and watchful sleep operating modes of ONU devices. A comparison related to the optical transceiver activity states, transition times, power savings, and data traffic handling for these ONU device operating modes is presented in Table 8.

### 4.1. Sleep ONU Operating Modes

Sleep mode and doze operating mode of optical network devices represent widely adopted energy-saving strategies for ONU devices, designed to minimize ONU power consumption during periods of low traffic activity [3,41]. Sleep and doze modes have been specified in Annex E of [ITU-T G.984.3] for GPONs and can be found in practical implementations since 2014 onwards. Also, sleep and doze operating modes have been specified in clause 16 of ITU-T G.987.3 [6] for XG-PON since 2010. Doze and sleep operating modes are related to each other, and both of them need to be simultaneously implemented to be compliant with the appropriate recommendations. The main characteristics of ONU sleep and doze operating modes are presented in Table 7, while a performance comparison of these operating modes in terms of their differences in ONU device operation, energy savings, latency, and suitability for traffic handling is presented in Table 8. Sleep mode enables deactivation of the optical receiver and all non-key ONU functions, while only the operation of activity detection is left in the active state when the ONU device is not in use. This operating mode includes two variations of sleep states, which include basic sleep and deep sleep operating modes.

In basic sleep operating mode, all functions except the basic ones are turned off, and reactivation is possible through the reception of a local signal or a set timer (Table 8). Basic sleep alternates between ONU idle and active operating states, allowing occasional data traffic transmission over the ONU device (such as for network browsing or message reception). The main advantage of this sleep mode is the possibility of obtaining significant energy savings due to more extended periods of ONU operating inactivity, but its disadvantage is the delay in establishing ONU synchronization with OLT (Table 8). The OLT device uses ONU management and control interface (OMCI) frames or Physical Layer Operations, Administration, and Maintenance (PLOAM) messages to manage, configure, and control the operating mode of the ONU device. The TDM PONs use sleep-mode technology, which enables a significant reduction in EC during inactive periods [3,41,42].

The deep sleep ONU operating mode is a power-saving state designed to maximize EE by shutting down most or all of the ONU’s active components during prolonged idle periods (Table 7). More specifically, in the deep sleep mode of operation, the ONU completely powers off key components, such as the optical transmitter (Tx), optical receiver (Rx), media access control (MAC), and physical layer (PHY) processing units, and sometimes even memory and CPU subsystems (Table 8). The deep sleep mode of ONU operation enables maximum power saving. It is based on switching off all ONU functions and services except activity detection. The consequence of switching off the ONU is the loss of incoming downstream signaling and data traffic. The ONU may switch on by receiving some local stimulus or after the expiration of a timer maintained by the ONU. In order to avoid unnecessary alarms, the OLT needs to be informed when each ONU transitions into the deep sleep mode. The OLT in the ONU deep sleep mode of operation needs to treat the absence of upstream traffic as normal, while discarding or continuing to send downstream traffic and providing upstream allocations.

#### 4.1.1. ONU Doze Operating Mode

Doze mode is a power-saving state in which the ONU reduces or disables its optical transmitter (and sometimes other internal components) during idle uplink periods, while still keeping the optical receiver active to maintain synchronization and receive downstream traffic from the OLT (Table 8). The local stimulus or the OLT request in doze mode can instantaneously power on the ONU. Unlike deep sleep modes, doze mode keeps the receiver active to avoid reinitialization delays. In comparison with the deep sleep mode of operation, the ONU can wake up more quickly (on the order of μs to ms) from the doze mode to resume upstream transmission (Table 8). The doze ONU operating mode is often implemented alongside sleep and cyclic sleep modes for finer energy management.

Although the energy saving in doze operating mode is lower in comparison with that in sleep mode, its operating mode transition impact on data traffic latency is minimal, which makes it suitable for shorter periods of data traffic inactivity (Table 8). The combination of sleep and doze operating modes, along with adaptive timers, enables a balance of ensuring the improvement of network EE of ONU devices while providing QoS. Therefore, sleep operating mode enables more energy savings with increased latency, while doze mode provides faster operating readiness of the ONU device with less EE improvement (Table 8).

### 4.2. ONU Cyclic Sleep Operating Mode

An ONU in the cyclic sleep mode periodically alternates between sleep and active periods, and this operating mode represents a variant of the ONU sleep mode (Table 7). During the sleep state, the optical transceiver and all non-essential functions are switched off, while activity detection and timing remain active. During the active period, the optical transceiver and required supporting functions are fully powered (Table 8).

The OLT uses a unicast or broadcast sleep PLOAM message for controlling the transitions among active and sleep periods, and this transition needs to be synchronized by all the ONUs operating in the cyclic sleep mode. After receiving the sleep PLOAM massage, an ONU in the cyclic sleep mode starts the sleep period. In comparison with the doze operating mode, the ONU in the cyclic sleep operating mode does not maintain all clocks (Table 8). During the sleep period, the ONU maintains only a clock that generates a signal for powering the receiver (Rx) in advance of the scheduled activation time frame. The OLT saves the incoming downstream data traffic for the ONUs in the sleep period and delivers it to the ONU when the ONU changes its state to active. This guarantees that the downstream traffic will be delivered to the ONU while it is in the sleep state.

**Figure 3:**
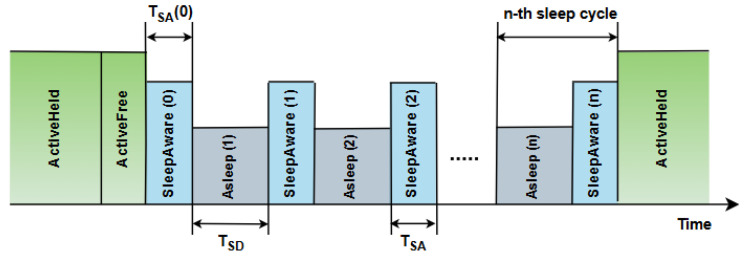
Timing diagram for ONU power management within the fixed-cycle sleep mode (FCSM) mechanism [43].

Figure 3 presents an example of the time diagram for ONU power management within the fixed-cycle sleep mode (FCSM) mechanism [43]. Figure 3 presents the system’s initial state, which is the active state (ActiveHeld), where the ONU is fully operating and performing all functions with maximum power consumption. After that, the ONU transitions to the ActiveFree state, which has lower power requirements, while preparing to enter sleep mode (Figure 3). After the active phase, the ONU enters SleepAware mode, which is a transitional state between the active and sleep (Asleep) modes. During the Asleep phase, the ONU device is in the state with the highest reduction in power consumption. During these periods, minimal ONU device activity occurs, while maintaining the ability to quickly respond to network requests. The ONU can make periodic transitions between SleepAware and Asleep states for a number of sleep cycles (Figure 3). According to Figure 3, the fixed duration of each phase is defined by parameters such as time to transition to the sleep state ($T_{SD}$) and time to transition from the sleep state ($T_{SA}$). At the end of the cycle, the unit exits the sleep mode and transitions back to ActiveHeld operating mode to perform full functionality [43].

### 4.3. Watchful Sleep Operating Mode

Energy efficiency of sleep operating mode for optical networks is further advanced with the introduction of watchful sleep (Wsleep) operating mode (Table 7). Watchful sleep mode has started to predominate in practical implementations since 2016, both for the NG-PON2 standardized in ITU-T G.989.3 [44] and the XG(S)-PON standardized in ITU-T G.9807.1 [45]. Watchful sleep operating mode has become the standard that combines the doze and sleep operating modes into one operating mode. It serves as an intermediate mode between doze and cyclic sleep ONU operating modes, which optimizes EE while maintaining fast downstream responsiveness (Table 8). However, the doze and sleep modes are maintained in practical implementations in cases when compatibility with older ONU generations was necessary. For example, to simultaneously connect the combination of XG-PON and XGS-PON ONUs, XG(S)-PON OLTs have to simultaneously maintain doze mode and cyclic sleep mode of operation.

**Figure 4:**
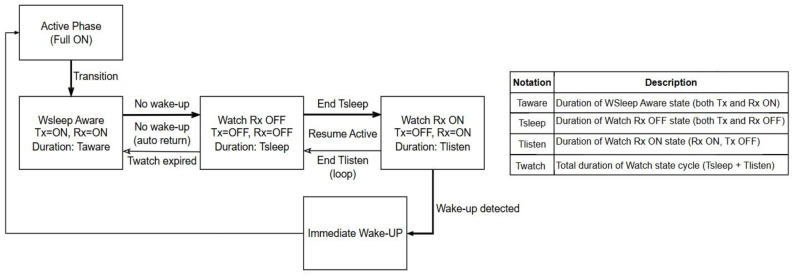
State transitions during the watchful sleep mode for ONU power saving [46].

Watchful sleep is a power-saving operating mode for an ONU device, where the optical transmitter is turned off but the optical receiver remains active, allowing the ONU to continuously monitor downstream traffic without transmitting (Table 7). In PONs, the watchful sleep (Wsleep) operating mode is composed of three energy-saving operating states: WSleep Aware, Watch Rx OFF, and Watch Rx ON. Figure 4 depicts an example of the cyclic transitions between these states as part of the watchful sleep mechanism. Upon transitioning from the active phase, the ONU enters the Wsleep Aware state, where both the transmitter and receiver are active for a defined duration T_aware_ (Figure 4). If no wake-up signal is received during this period, the ONU transitions into the Watch Rx OFF state, in which both the transmitter and receiver are deactivated for a duration T_sleep_. Following this, the device proceeds to the Watch Rx ON state, where only the receiver is active while the transmitter remains in the off state (Table 8), lasting for a duration T_listen_ (Figure 4). These two states alternate cyclically, forming the watch phase, and this cycle continues until the cumulative duration T_watch_ expires (equal to the sum of T_sleep_ and T_listen_ intervals). If no wake-up signal is received within this total watch duration, the ONU re-enters the WSleep Aware state for a second T_aware_ interval (Figure 4). If the wake-up signal remains undetected after this repetition, the ONU returns to the active phase automatically. Conversely, if a wake-up signal is detected during any of the Watch Rx ON intervals, the ONU immediately exits the energy-saving mode and resumes active operation (Figure 4).

Therefore, in the watchful sleep operating mode, the ONU’s transmitter is disabled to save energy, and the optical receiver stays switched on, “listening” for downstream messages from the OLT (Table 8). Thus, unlike the deep sleep operating mode, there is no need to periodically power up the optical receiver just to check for downstream frames. If downstream traffic is detected or a scheduled upstream transmission is needed, the ONU quickly wakes up and reactivates its transmitter (Table 8). This mode avoids the overhead of full sleep cycles (e.g., re-synchronization or wake-up signaling), since it maintains the optical receiver in an active state for quick response. This eliminates the penalties of frequent wake-ups, since the watchful sleep operating mode avoids the energy and time costs of the full wake-up procedures that occur in deeper sleep modes. The watchful sleep operating mode is suitable for scenarios with low upstream traffic and enables the exchange of occasional downstream control messages. The EE improvements that the watchful sleep mode of operation brings are seen in reduced optical transmitter power use (Table 7 and Table 8). This is because keeping the optical transmitter switched off during the idle upstream period enables energy saving, especially in low-load or bursty upstream traffic scenarios.

#### 4.3.1. Wake-Up Strategies of Watchful Sleep Operating Mode

This watchful sleep operating mode considers two ONU wake-up strategies, known as Delayed Wake-Up (DWU) and Immediate Wake-Up (IWU) [46]. The watchful sleep mode and DWU strategy are ONU energy-saving techniques standardized in ITU-T G.987 [30] for XG-PON. The role of the DWU strategy is to extend the energy-saving state of the ONU by delaying the full wake-up of the ONU, even after the OLT has signaled to the ONU that data is available for transmission. The DWU helps the ONU remain longer in sleep mode of operation, while the OLT schedules upstream or downstream traffic from or to the ONU, respectively. The DWU enables ONU devices to achieve up to 80% energy savings with Time-Delayed Wake-Up (DWU), especially in low-traffic-load conditions [46]. Besides improving EE, DWU prevents the ONU from rapidly switching between sleep and active states for short or bursty traffic, which has an impact on the improvement of the ONU device’s longevity.

Therefore, the DWU strategy enhances the watchful sleep operating mode by introducing a smart delay mechanism that allows the ONU to stay in a low-power state slightly longer, even when traffic is pending for transmission by the ONU. This watchful sleep and DWU enable the ONU to periodically enter low-power states while maintaining synchronization with the OLT and strategically performing DWU to reduce unnecessary EC. The OLT has a critical role in coordinating and signaling the ONUs to enter or exit sleep modes via OMCI frames and PLOAM messages. Thus, watchful sleep techniques are generally not applicable to OLTs, as they must be active to control multiple ONUs.

Besides DWU, another wake-up strategy in watchful sleep operating modes is IWU (Figure 4). Implementation of the IWU strategy results in ONU waking up immediately from watchful sleep mode, as soon as it detects that the OLT has scheduled data transmission (e.g., via a grant or a control message). Table 9 presents a comparison of these two sleep strategies, illustrating how they differ and in which implementation scenarios each method is most effectively applied. The IWU strategy, in comparison with DWU, results in lower energy savings, because the ONU exits sleep mode more frequently and quickly and has a higher responsiveness, but at the cost of increased power consumption.

As presented in Table 9, each method offers distinct operating advantages, making them suitable for different deployment scenarios, depending on whether activity responsiveness or EE is the primary objective. While IWU focuses on providing a quick response when traffic arrives, DWU aims to save more energy by delaying the activation of the device. The greater energy savings of DWU compared to IWU due to avoiding frequent or premature ONU wake-ups are at the cost of a slightly increased latency, which is still acceptable for most non-real-time applications (file downloads, web browsing, etc.). Additionally, DWU has an indirect impact on improving OLT energy consumption, since OLTs are always active, and fewer ONU wake-ups reduce OLT signaling and scheduling workload. This contributes to slightly reducing OLT power consumption and improving overall system EE. Modern ONUs may support both strategies (DWU and IWU), and switching between them depends on the traffic type, QoS requirements, and network policy in terms of what the network prioritizes, whether it is delivering fast service or achieving higher EE over time.

#### 4.3.2. Variants of Adaptive Management of Sleep Modes for Enhancing EE

As presented in the previous section, cyclic sleep mode (CSM) and watchful sleep mode (WSM), as two important approaches to achieving improved ONU EE, are examined. The fundamental difference between these methods lies in how the ONU receiver is managed during sleep mode. CSM completely disables the optical receiver activity, whereas WSM allows periodic transitions between sleep and partial activity, which contributes to reducing data transmission delays and ensuring better QoS. However, in practice, challenges related to managing symmetrical time intervals for switching the ONU optical receiver on and off exist. These challenges result in the improper adjustment of sleep duration based on network traffic load, which negatively impacts the improvement of the overall ONU EE. Adaptive Watch Sleep Mode (AWSM) is introduced as an enhanced version of WSM to address these limitations.

**Figure 5:**
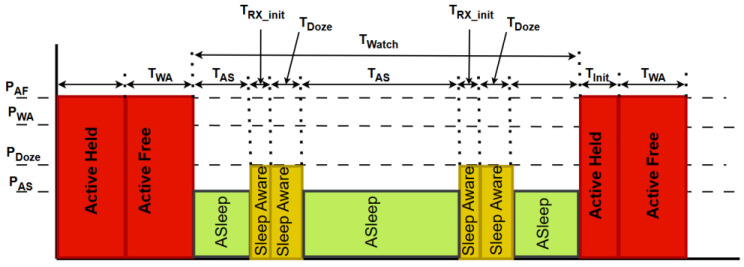
Different levels of EC and durations of ONU retention states for the AWSM ONU scheduling mechanism [47].

The AWSM applies to a transition mechanism comparable to WSM but incorporates modifications in the monitoring phase management. This approach relies on an asymmetrical adjustment of ONU receiver activation and deactivation durations, aiming to improve ONU EE while maintaining QoS. Figure 5 presents an example of ONU activity scheduling based on AWSM, demonstrating how ONU transitions between high activity, monitoring, and sleep states contribute to optimizing EC [47].

The example presented in Figure 5 for the AWSM ONU management scheme enables the optimization of EC in optical ONU devices through intelligent transitions between different states. Power consumption in the Active Full ($P_{AF}$) state (ActiveHeld) represents the power consumption of a completely active ONU, utilizing the highest energy level for the full functionality of the ONU device. The power consumption in the Watching Active ($P_{WA}$) state (ActiveFree) reduces EC while maintaining essential network monitoring (Figure 5). The power consumption in the doze ($P_{Doze}$) state (SleepAware) and power consumption in active sleep ($P_{AS}$) state (Asleep) enables additional energy savings in periods of no network activity (T_watch_), also ensuring the ONU device is ready to return to active state mode quickly. Compared to the FCSM of operation presented in Figure 3, the primary goal is to extend the sleep duration to achieve maximum energy savings, while the duration of active states ($T_{WA}$ and $T_{Init}$) is reduced during low-traffic periods. The AWSM of ONU operation improves state transitions by adjusting the ONU optical receiver initialization time ($T_{RX\_Init}$) and the duration of the doze phase ($T_{DOZE}$). According to simulations presented in [10], which are conducted for an XG-PON with 32 ONUs, AWSM achieves up to 71% energy savings under low-network-activity conditions, which is 9% more than the savings achieved by WSM [47].

## 5. Resource Management Techniques for Optimizing the Energy Consumption of ONU and OLT Devices

This section analyzes advanced resource management strategies for ONU and OLT devices, which contribute to the improvement of ONU and OLT EE. The analyzed strategies include resource management for joint optimization of OLT and ONU energy consumption through dynamic transition among different operating modes of ONU and OLT devices. Also, in this section, the resource management approaches for reducing EC at the level of the individual ONU and OLT devices are analyzed.

### 5.1. Resource Management for Joint Optimization of OLT and ONU Energy Consumption

**Figure 6:**
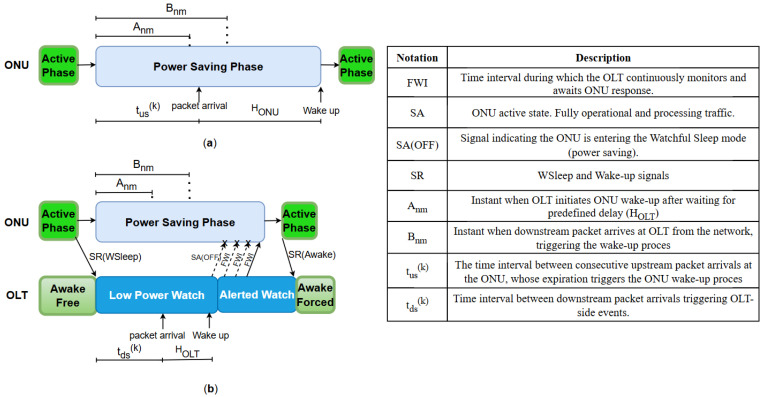
Power-saving phase control and wake-up mechanisms in ONU–OLT interaction: (**a**) ONU-initiated wake-up based on upstream packet arrival, (**b**) OLT-initiated wake-up based on downstream traffic or timer expiry [46].

Figure 6 presents an example of a standardized model of ONU and OLT device activity management in phases of energy saving and active operation, where wake-up occurrence can be initiated by the ONU device (Figure 6a) itself or by the OLT device (Figure 6b) [46]. Figure 4 visualizes the transition process between energy-saving and the active states in watchful sleep mode for the DWU mechanism. Table 10 presents an explanation of operating phases of ONU and OLT devices, providing an overview of their roles during the energy management process in PONs.

The presented model in Figure 6a highlights how ONU shifts between the active and power-saving phases. The ONU-initiated wake-up scenario (presented in Figure 6a) begins during the power-saving phase. When the ONU is in one of the low-power states (Watch Rx OFF or Watch Rx ON) and an upstream packet arrives at the ONU at instant A_nm_, the wake-up procedure does not commence immediately. Instead, the ONU waits for a predefined delay ($H_{ONU}$) before performing the transition from the power-saving phase to the active phase (Figure 6a). After $H_{ONU}$ expires, the ONU wakes up fully, transitioning to the active state to handle network traffic.

Figure 6b shows the ONU transitions between active and power-saving phases, while the OLT manages low-power states (Low-Power Watch, Alerted Watch) and wake-up signals (SR and SA) to optimize ONU EE and response time based on network traffic conditions. In the case of the OLT-initiated wake-up scenario (Figure 6b), the wake-up procedure is triggered by the OLT upon the arrival of downstream traffic. Initially, the OLT resides in the Awake Free state while the ONU is fully active, and no permission is yet granted for ONU power-saving transitions (Figure 6b). Subsequently, the ONU informs the OLT with an SR (WSleep) message about entry into the power-saving state, and the OLT consequently changes operating state to the low-power watch state, in which it expects periodic messages from the ONU while buffering incoming downstream traffic. When a downstream packet arrives at an instant $A_{nm}$ (Figure 6b), the OLT waits for the predefined delay ($H_{OLT}$) before initiating the wake-up procedure by sending the SA (OFF) message to the ONU (Figure 6b). After $H_{OLT}$ expires, at instant $B_{nm}$, the OLT transitions to the Alerted Watch state and sends repeated wake-up signals (in regular Full Watch Interval (FWI)) during subsequent allocation cycles to trigger the ONU’s return to the active phase. Once the ONU wakes up, it sends the SR (Awake) message to the OLT and informs the OLT about its state transition (Figure 6b). After the ONU acknowledges the wake-up to the OLT by sending an SR (Awake) message and confirms its active state (Figure 6b), the OLT transitions back into the Awake Forced state, resuming normal network operation. This adaptive power management strategy is crucial in reducing EC while maintaining seamless network operations, making PONs more efficient and sustainable.

#### 5.1.1. Energy Management Models of OLT/ONU Devices Based on the Standardized Watchful Sleep Operating Mode

**Figure 7:**
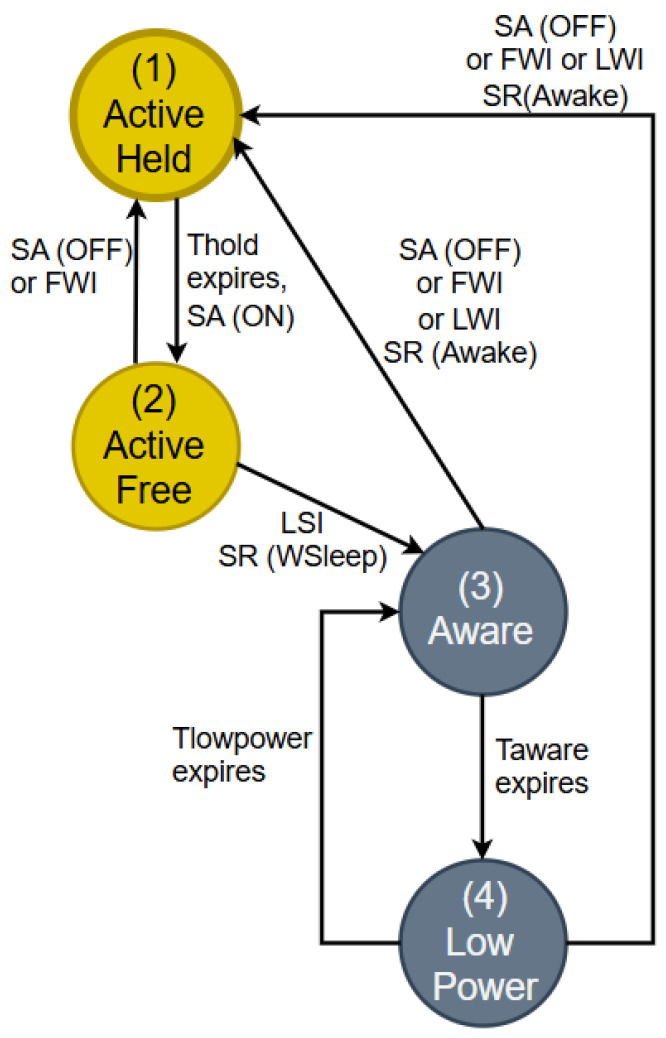
The state transition diagram for ONU device EE management in watchful sleep mode of operation [8].

Another example of the latest standardized ONU device watchful sleep operating mode defined in the ITU-T G Suppl. 45 recommendation from 2022 is presented in Figure 7 [8]. The diagram presented in Figure 7 illustrates how the ONU device can optimize EC in the watchful sleep operating mode by shifting from one operating state to another, according to timing conditions and network demands [8].

In the Active Held state (State 1), the device works in a fully active mode (known as Sleep Allowance (SA) (ON)) and provides complete functionality for network traffic (Figure 7). After the Hold Time (T_hold_) expires, the device switches to the Active Free state (State 2) when SA (OFF) is activated or receives a wakeup signal via the OLT Frame Wakeup Indicator (FWI). In this state, the device remains active, but with lower energy consumption, contributing to energy optimization (the relationship between watchful sleep mode state transitions can be analyzed by comparing the diagrams in Figure 3 and operating states in Figure 7). If the Low Sleep Indicator (LSI) is active during the Active Free state, the device switches to the Aware state via Initiate Sleep Request (ISR) (W_sleep_), initializing the sleep mode (Figure 3 and Figure 7). In the Aware state (State 3), the device maintains “awareness” of network traffic with minimal activity. This state enables network monitoring without full activation, which results in reduced EC (Figure 3). When Awareness Time (T_aware_) expires, the device switches to the low-power state (4) (Figure 7), where it enters the mode of minimum energy consumption. In this state, the device maintains only essential functions in order to ensure significant reductions in energy consumption. However, this state still ensures the readiness for reactivation when the OLT FWI or Link Wakeup Indicator (LWI) signals occur (Figure 7). This allows the device to return to Active Held states to support network traffic at full capacity again (Figure 3 and Figure 7).

#### 5.1.2. DBA Method for Improving Optical Network EE

According to [48], dynamic bandwidth allocation (DBA) represents an indispensable method for optimizing the EE of PONs (e.g., GPON, XG-PON) and is a standard feature implemented in contemporary ONU and OLT devices. DBA in optical networks refers to a method where the OLT dynamically adjusts bandwidth assignments to each ONU according to their real-time bandwidth requirements. Each ONU periodically communicates its traffic load and queue occupancy to the OLT by sending status reports. The OLT collects and analyzes bandwidth requests from all connected ONUs and determines bandwidth allocation based on ONU demands, defined Quality of Service (QoS) policies, and network traffic conditions. In Time Division Multiplexing (TDM)-based optical networks, the OLT sends downstream grant messages to ONUs indicating precisely when and for how long each ONU may transmit data upstream in allocated timeslots, avoiding collisions with other ONUs.

The DBA method significantly contributes to improving optical network EE by enabling efficient ONU sleep (such as watchful or deep sleep) mode of operation and grouping ONU data upstream transmissions into bursts. This allows the ONU to remain in low-power (sleep) mode during idle periods of upstream transmission and transition (waking) from sleep to active mode only when a bandwidth for upstream transmission is granted and allocated by the OLT. Also, the DBA approach reduces the overall ONU active (fully powered) time, since a specific ONU is active only during assigned upstream transmission intervals, and the less active time of the ONU device directly reduces overall ONU energy consumption. Thus, the DBA method enables ONUs to match the duration of their upstream transmission activity periods with instantaneous bandwidth requirements, thereby reducing the ONU’s overall EC and the transition time of the ONU device from the idle state to the active state.

In Wavelength Division Multiplexing (WDM)-based networks, each ONU typically has a dedicated wavelength for upstream and downstream communication, and no inter-ONU time slot competition on a single wavelength is needed. DBA dynamically manages bandwidth per wavelength in response to ONU demands and allows the ONU or OLT to enter sleep or low-power modes when lower bandwidth demands occur. In addition, DBA can dynamically select lower-power modulation schemes during low-traffic periods, and such adaptability directly reduces ONU power consumption when higher bandwidth or complex modulation is not necessary. Thus, ONUs and OLTs periodically use sleep or low-power modes, which can effectively reduce devices’ network power consumption. The EE of these devices is related to the duration of the sleep cycle, with more extended sleep periods increasing efficiency. According to [49], the relationship between the power consumption of the OLT PON line card and the ONU device and the duration of sleep and wake cycles is analyzed, demonstrating that power consumption gradually decreases as the cycle length increases.

Implementation of the DBA method (algorithm) in practice confirms that bandwidth management methods (algorithms) are essential for optimizing network resources and improving EE of optical networks. These methods manage the distribution of optical network resources between the ONU and OLT, intending to reduce delay, ensure appropriate resource distribution, and optimize network energy consumption. Table 11 presents an overview of performance capabilities with a focus on the impact on OLT and ONU EE of some well-known DBA algorithms, which include Grant Independent Allocation with Non-Overlapping Transmission (GIANT), Hybrid Reservation and Allocation (HYRA), Modified Max-Min Fair, and static allocation (which is not a dynamic algorithm and is used for a baseline for comparison).

The analyzed DBA methods in PONs presented in Table 11 show that each bandwidth scheduling approach has specific advantages and challenges [50]. The GIANT algorithm offers optimal resource allocation, but results in generating more “idle” frames that contain no actual payload of user data and that are generated by the OLT to maintain continuous downstream synchronization and timing of the ONU, even in periods of low or zero user traffic. However, continuous generation of idle frames increases the OLT’s power consumption due to the always-active transmitter, and this limits the OLT’s ability to enter low-power modes, which affects overall energy efficiency. HYRA is a more energy-efficient DBA method with slightly higher latency. The Modified Max-Min Fair DBA method ensures fair bandwidth allocation but faces long delays and high energy inefficiency. Static allocation as a baseline DBA method is simple to implement, but produces significant energy losses due to non-optimized resource management. Hence, when choosing an appropriate DBA algorithm, it is necessary to carefully consider the specific requirements of the network, such as low-latency prioritization, energy saving, or fair bandwidth distribution [50].

### 5.2. Resource Management for Optimization of OLT Energy Consumption

In the literature, various strategies focused on advanced resource management methods for reducing the EC of OLT devices are examined [8]. An overview of methods for improving the EE of OLT devices is presented in Table 12. These methods include techniques such as automatic shutdown of inactive OLT device elements, implementation of the optical splitting ratio adjustment on OLT ports, and dynamic OLT port data rate adaptations.

According to [6], reducing the EC of OLT devices can be realized by disabling unused elements, such as inactive network line cards, optical transceivers (lasers, receivers), and network ports (Table 12). It is a technique that is also known as selective component deactivation or component-level power management. Powering down inactive elements reduces OLT EC by deactivating unused elements during periods of low or no traffic activity (Table 12). In addition, by reducing the number of active components, less heat is generated, and the cooling systems of the OLT device work less, saving power used by internal fans and external air-conditioning systems in the equipment room.

Therefore, selective deactivation of OLT components allows the system to intelligently match energy use to actual data traffic demand by turning off idle or underutilized elements. This improves optical network EE and reduces operating costs.

Another method, known as the OLT device port optical split ratio adjustment technique, can also enable energy savings of OLT devices (Table 12). Adjusting the optical split ratio allows active ONU devices to be redirected to a common OLT port, thereby turning off inactive OLT ports for additional energy savings. Therefore, during low network activity, the ONU devices can be redirected to the shared active OLT port(s), while other OLT ports having no or low data traffic can be temporarily disabled. This method in practice results in fewer active OLT ports, since a higher split ratio (e.g., increasing from 1:16 to 1:32) allows more ONUs to be served in PONs by fewer active OLT ports. This method dynamically controls the split ratio using optical switches and contributes to the overall reduction in EC [8].

**Figure 8:**
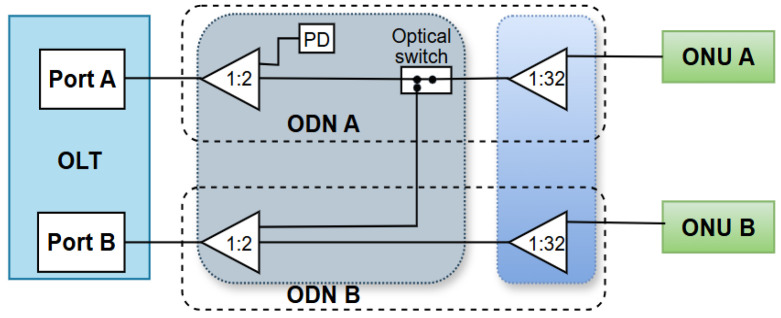
Architecture of an EE PON with a redundant protection mechanism [8].

Figure 8 presents an example of a system based on port optical split ratio adjustment. The system enables optimizing optical access network EC using passive splitters (1:2 and 1:32), a photodetector (PD), and an optical switch [8]. The system relies on redundancy and rational management of passive optical components. In order to save power during low traffic activity in ODN A and ODN B, the PD on ODN A monitors when the OLT A port is turned off and then controls the optical switch to combine ONU A and ONU B traffic to a common ODN B with a dynamic modification of the optical split ratio. Thus, the optical switch, in combination with the photodetector (PD), enables the activation or deactivation of the primary optical path that enables the connection of ONU A with the corresponding OLT port A over ODN B (Figure 5). The backup path remains in a standby state, and in case of low traffic activity on ODN A or failure of the primary path, the backup path is activated when necessary. Data traffic of the active ONU devices connected over the 1:32 optical splitter in ODN A is directed to a common OLT port B, while the unused OLT port A transits to a power-off state [8]. This contributes to reducing OLT EC under normal operating conditions.

Another method for improving OLT device EE is related to the dynamic adjustment of the OLT transmission data rate to reduce EC at low network load (Table 12). Dynamic OLT data rate adjustment involves scaling down the transmission speed (e.g., from 10 Gbps to 1 Gbps) during periods of low traffic demand, thereby reducing the EC of the OLT and associated optical/electrical components. Reduced data rates result in slower frequency clocks, lower bias (laser, drive) currents, and reduced power draw in line cards, transceivers (optical modules), and digital signal processors (DSPs). Some modern OLTs support energy-proportional behavior, in which power consumption scales with actual data throughput. Dynamic data rate adjustment at the OLT reduces EC by scaling down electrical and optical activity in line with data traffic variations. This enables the OLT to operate in a more energy-efficient mode during low-load periods, without compromising QoS.

### 5.3. Resource Management for Optimization of ONU Energy Consumption

Although the share of ONU devices’ EC in the total EC of households or buildings remains relatively small, the increase in their complexity and the global number of ONU devices requires constant improvements in ONU device EE [8]. The increased ONU device EC due to the increase in the global number of ONU devices is a consequence of the continuous increase in the penetration of different Fiber-to-the-x (FTTx) technologies. The increased complexity and, consequently, EC of modern ONU devices is reflected in integrating additional functionalities into ONU devices, such as a wireless local area network (WLAN) communication module that supports IEEE 802.11x and Wi-Fi 5, 6, and 7 standards, and/or wired Ethernet technology with data rates of up to 10 Gbit/s [4]. As ONU devices become more advanced through support of Ethernet connections and, for some models, Wireless Fidelity (Wi-Fi) standards, it becomes increasingly important to manage their energy use intelligently.

However, these intelligent management methods dedicated to optimizing ONU EE must be developed in such a way as not to compromise users’ QoS. Table 13 presents characteristics of some of the most important methods for reducing the power consumption of ONU devices. Methods include reducing the ONU port data rate, reducing the number of ONU active antennas, and using the ONU power-shedding method. All these methods are designed for dynamic ONU resource adaptation, ensuring automatic alignment with demands for the transfer of network data traffic.

#### 5.3.1. Reducing ONU Port Data Rate

Table 14 presents the average instantaneous power consumption of a typical single-port active ONU device, without routing, gateway, or WLAN functionalities, operating in different optical access network types with corresponding downstream (DS) and upstream (US) data transmission rates [43]. According to Table 14, the ONU EC increases for cases where the ONU is operating in optical access networks that ensure higher transmission data rates. Table 14 also indicates that an ONU operating in optical access networks like Fast Ethernet (FE) P-t-P produces the lowest transmission data rates and has the lowest average instantaneous power consumption. More advanced optical network types, such as GPON, 10G EPON, and XG-PON, enable higher transmission data rates, along with significantly higher EC of the ONU device.

According to Table 14, lower data transmission rates of ONU devices result in lower average power consumption of ONU devices. Thus, reducing the data rate of the ONU port (e.g., from 1 Gbps to 100 Mbps) can contribute to improving EE of ONUs, especially in low-traffic or idle conditions (Table 13). This improvement of ONU EE is a consequence of reducing the port speed, which results in reduced power draw of the ONU PHY and the MAC interface and a lowering of switching frequency in the optical transceiver electronics. Lowering the speed of PHY ONU component operation (by implementing slower clock speeds, reducing chip frequency, lowering operating voltages, and reducing the number of active processing units) also reduces thermal output, which contributes to improving the ONU passive cooling efficiency, extending ONU device lifetime, and lowering overall ONU energy needs.

Therefore, adjusting the ONU port data rate according to data traffic intensity, which is also known as the adaptive link rate (ALR) concept, contributes to lowering ONU power consumption and consequently enables more energy-efficient ONU operation without disrupting user experience (Table 13). Research has been performed in terms of improving ONU EE by integrating the ALR with the cyclic sleep mode. This approach represents a hybrid solution that adjusts the sleep cycle duration and port link data speed based on data traffic conditions of the ONU port. According to [3], the ALR method in hybrid solutions enables dynamic switching of the downstream link data rate between 1 Gbps and 10 Gbps, depending on the amount of network traffic. Thus, it is proven that the ALR method can work with the cyclic sleep mode to further reduce ONU EC during periods of reduced data traffic activity.

**Figure 9:**
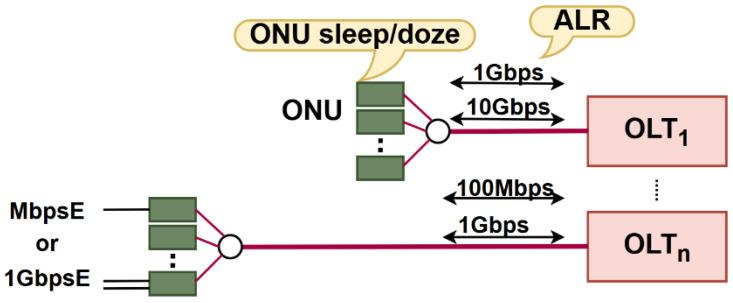
Application of ALR with sleep/doze modes for EE in OANs [16].

In Ref. [16], a hybrid ONU operating mode combining the sleep/doze mode and ALR in optical access networks is analyzed. Figure 9 presents energy-saving technologies applied in optical access networks that include a hybrid operating mode of the ONU device that combines sleep/doze modes and ALR [16]. In the first case, the ALR is implemented through parallel receivers in ONU devices with 1 Gbps and 10 Gbps capacities, enabling dynamic switching between these data rates within a single PON. In the second case, ALR adjusts the transmission data rate between 100 Mbps and 1 Gbps based on network load requirements (Figure 9). Therefore, such a hybrid approach is identified as a technology that adjusts the EC of the ONU device to the current data transfer rate and network load level. Such adaptation allows for optimizing the EE of the ONU, reducing unnecessary EC during low activity.

#### 5.3.2. Reducing the Number of Active WLAN Antennas

ONU devices differ among device manufacturers and vendors not only in the supported optical network type but also in the capability of supporting or not supporting WLAN connectivity. For ONU devices that have the option of supporting WLAN connectivity, reducing the number of active multiple-input multiple-output (MIMO) WLAN (Wi-Fi) RF chains and corresponding antennas in an ONU during periods of low user demand, idle operation, or limited coverage can contribute to improving ONU EE (Table 13). However, dynamic scheduling of the activity of ONU RF antenna chains according to data traffic variations does not contribute to the optimization of ONU network EC in the optical domain, but rather is related to the optimization of ONU EE in the wireless domain.

Since each active ONU device Wi-Fi antenna requires a separate radio frequency (RF) chain (which includes RF front-end, amplifier, filters, and power amplifiers (PAs) consuming significant energy during transmission), switching off one or more MIMO antennas (e.g., from 4 × 4 MIMO to 1 × 1 MIMO) reduces RF signal generation and lowers RF signal transmit power (Table 13). Also, a lower number of ONU active MIMO RF antenna chains leads to simpler modulation/demodulation of the RF signal and less signal processing for wireless signal beamforming, diversity, or spatial multiplexing. All this contributes to decreasing the overall power consumption of the ONU WLAN subsystem (Table 13). In addition, modern ONUs can implement traffic-aware or user-aware antenna control. This is based on the concept that if only one user device is connected to the ONU or traffic is minimal, the number of active antennas and corresponding RF chains is reduced to one, and when more devices join or high throughput is needed (e.g., video streaming), the additional MIMO RF chains and corresponding antennas are dynamically reactivated. This approach allows real-time balancing between performance and energy savings.

#### 5.3.3. ONU Power-Shedding Method

In Ref. [41], a power-shedding method is also discussed. Power shedding is the capability of an ONU device to reduce power consumption during alternating current (AC) power outages (Table 13). It is based on the assumption that the ONU is connected with the energy source that contains a battery supply backup. Upon reception of the information about the loss of the AC power source and transition to another (such as direct current (DC)) power source, the ONU may reduce power consumption by switching off specific ONU interfaces. The concept involves turning off non-essential components of the ONU device while maintaining the essential network connection (Table 13). However, the contribution of the power-shedding method to energy savings is limited only to periods of AC power outages.

### 5.4. Advanced Technologies for Improving EE of Future OANs

Beyond the presented traditional resource management techniques for optimizing the EC of ONU and OLT devices, additional technologies are expected to emerge in the future as promising techniques that can contribute to the reduction in OAN EC. An example of such technology is the implementation of the optical network coding (ONC) technique in OANs.

Network-coding-enabled optical networks are advanced optical communication networks (e.g., Dense Wavelength Division Multiplexing (DWDM), PON, or elastic optical network) that use algebraic data encoding. The ONC technique enables multiple data streams to be simultaneously encoded together (e.g., using OR, XOR, or linear combination operations), and only a single coded data stream is transmitted. This is contrary to the traditional data stream transmission approaches, which are based on the transmission of multiple separate data streams. At the receiver, the ONC technique enables the original data streams to be decoded from the combined encoded data stream. The ONC concept integrates network coding techniques at various layers, such as at the electrical layer before modulation, or at the optical layer (which is still in an experimental phase).

Studies show that applying ONC in core/metropolitan Internet Protocol over Wavelength Division Multiplexing (IP/WDM) networks achieves a reduction in the number of simultaneously active optical resources for a given QoS. This yields measurable energy savings of up to ~37% in ring topologies and ~23% in typical topologies under 1 + 1 optical protection, and ~18% in bypass or ~28% in non-bypass optical network topologies at 400 Gb/s data rates [51,52]. Also, studies show that ONC contributes to fewer retransmissions and fewer network reconfigurations, all of which can contribute to improving optical network EE. While practical deployment of ONC techniques is still limited to backbone and core/metropolitan optical networks, the concept has strong implementation potential in OANs for improving network EE.

ONC can improve network EE in passive OANs through multicast stream encoding, fewer active ONU waking-up events, protection with coding (instead of using optical backup paths), and reduced OLT buffering and scheduling complexity. By implementing multicast stream encoding, instead of sending duplicate copies of the same data (e.g., video) to different ONUs, the OLT can send coded multicast packets, and ONUs decode their own content from shared data. This contributes to reducing redundant transmission at different wavelengths, which consequently lowers OLT EC. Additionally, application of the ONC technique can result in fewer active ONU waking-up events, which enables multiple ONUs to remain in sleep mode longer by decoding received encoded data from shared coded streams, rather than frequently waking up for separate receptions. Consequently, fewer active ONU waking-up events save ONU power consumption during idle times. In addition, performing the optical protection with the ONC technique allows PONs to handle link failures via coded redundancy, instead of activating separate optical backup paths. This results in fewer standby ONUs or OLT ports that remain powered in PONs. Also, the implementation of the ONC technique can improve network EE in passive OANs by combining multiple upstream flows into joint coded streams, which enables the OLT to perform less frequent scheduling and buffer management. This consequently results in lower processing EC at the OLT.

Furthermore, ONC can improve network EE in active OANs through reduced active switch port usage, energy-efficient multicast transmission, load balancing with optical-coded flows, and enabling optical network path survivability with fewer active backup paths. ONC can combine data streams into fewer flows, allowing some switch ports in active OANs to be deactivated or enter low-power mode. Thus, reduced active switch port usage reduces switching power consumption in remote nodes. Also, implementing ONC means that instead of duplicating traffic for each user at powered network switches, network-coded multicast sends one coded flow. Such multicasts ensure less processing and fewer forwarding operations per multicast group, which improves network EE. Additionally, ONC can enable AONs’ load balancing with coded flows, which further contributes to better load distribution across fewer active links. Better flow distribution among access links ensures more idle links and ports that can be in the sleep state, which contributes to saving power. Finally, due to the often required active backup links in AONs, ONC can replace this requirement with coded redundancy, avoiding the need for fully powered backup switches. This can contribute to decreasing energy used for redundant infrastructure.

Therefore, implementation of the ONC technique in both access PONs and AONs has high unexploited potential for improving the EE of OANs by minimizing the number of active transmissions, reducing data duplication (redundant data paths), and optimizing network resource (equipment) utilization and activity. The implementation of the ONC technique can be a new technical concept for reducing the hardware footprint and energy per delivered bit in OANs.

## 6. Analysis of Global PON and AON FTTH Network EC

This section aims to present a deeper analysis of how the increase in the number of FTTH network subscribers impacts the optical access network EE on a global scale. The analysis is based on the technical specifications and power consumption declarations of OLT and ONU devices of one prominent manufacturer (the Slovenian company Kontron d.o.o. (Kranj, Slovenia), which is a member of the Austrian Kontron AG group, previously known as the company Iskratel d.o.o.). These devices are selected as typical representatives of optical network devices used in practical implementations and thus serve as example devices in the presented analyses. Additionally, this section also presents EE analyses of OLT and ONU devices, a structured methodology for calculating optical network EE, and an overview of the global EE trends related to two types of FTTH networks: point-to-point (P-t-P) and GPON FTTH networks. Thus, this section provides a comprehensive evaluation of EC metrics and EE calculations, aiming to give a broader perspective on how FTTH networks contribute to sustainable energy use on a global scale.

### 6.1. Methodology for Calculating Optical EE and Network Traffic Volume

This section outlines the methodology used to evaluate the EE of optical access networks. According to [53], optical access networks are significant energy consumers, especially during early-stage deployment. However, network EE can be improved with an increase in the transmission of user data volumes (number of users) and the adoption of power-saving mechanisms, such as putting part or complete optical network elements in sleep modes. Due to its practicality and relevance, the energy per transmitted bit $\left(E_{bit}\right)\;$is selected as the metric for evaluating and comparing the EE of FTTH GPONs and P-t-P networks in the analyzed deployment scenarios. It quantifies the amount of energy required to transmit a single bit of data across the network infrastructure and is particularly suitable for comparing large-scale FTTH deployments under varying load and configuration scenarios. As defined in [53], energy per transmitted bit ($E_{bit}$) is calculated using the ratio between the total power consumption of the optical access network (or network device) and the effective data rate (throughput) in the optical access network. Energy per transmitted bit can be expressed as

(1) $$E_{bit}=\frac{P_{total}}{R_{total}}\;\left[\frac{J}{bit}\right]$$

As noted in [42], typical values for energy per bit ($E_{bit})$ in long-range optical access systems are around 1.1 nJ/bit. In addition, total network traffic can be calculated as follows:

(2) $$R_{total}=N_{users}\cdot R_{users}$$

where $N_{users}$ represents the total number of users, and $R_{users}$ represents the average data rate (bps) per user. Equations (1) and (2) [53] form the computational foundation for the analyses of power and energy consumption values under different user distributions and FTTH architectures presented in subsequent subsections.

### 6.2. Analysis of EE, Capacity, and Operating Characteristics of ONU and OLT Devices

Table 15 and Table 16 present a detailed overview of the technical data for ONU/gateway (router) and OLT devices used in this analysis, respectively. The data presented in Table 15 and Table 16 indicate power consumption and EE characteristics that are typical for the current state of the art of ONU/gateway (router) and OLT devices. The data presented in Table 15 and Table 16 show values of the instantaneous power consumption of ONU/gateway (router) and OLT devices in different operating states, respectively. The presented instantaneous power consumption data are obtained through EC measurements that are performed in compliance with the ETSI EN 303 215 technical specifications and Code of Conduct on Energy Consumption of Broadband Equipment (CCECBE), issued by the European Commission’s Joint Research Centre [54]. The CCECBE sets out guidelines, principles, and power consumption targets for manufacturers and service providers to reduce the EC of devices such as broadband modems, ONU or OLT devices, routers, gateways (modem/router combinations), set-top boxes, and network extenders or repeaters. The technical specifications provide detailed information related to the energy requirements, particularly in “on” and “ready” modes (states) of operation (Table 15).

The “ready” operating state refers to an ONU/gateway (router) or OLT device’s standby operating mode, in which the device consumes minimal energy, as it is not actively transmitting data. This state corresponds to a low-power operating mode of ONU/gateway (router) or OLT device, where the equipment is not actively performing its primary functions and instead remains prepared to resume full operation quickly. In this state, certain device components may be powered down or operate at reduced capacity, resulting in lower EC compared to the “on” state of operation. In Table 15 and Table 16, the maximum power consumption thresholds in “on” and “ready” operating states set by CCECBE are presented for the analyzed ONU/gateway (router) and OLT device(s), respectively. Additionally, the “on” state represents a fully active operating mode of an ONU or OLT device and is characterized by higher instantaneous device power consumption compared with power consumption in the “ready” state (Table 15 and Table 16). In this state, all components of ONU or OLT devices are active and perform primary functions such as data transmission, routing, or signal processing.

To encourage energy-efficient designs, the CCECBE v9.0, Tier 2024, sets maximum power consumption limits for ONU/gateway (router) and OLT devices. Table 15 and Table 16 present the measured average instantaneous power consumption in the “on” state for these devices, along with their utilization percentage relative to the corresponding maximum allowable values. For ONU/gateway devices (Table 15), data for the “ready” operating state is also included, each compared to its respective limit. Compliance of ONU/gateway (router) and OLT device power consumption with the latest editions of the EU CCECBE EE thresholds for broadband equipment enables issuing EE certificates for specific optical devices. Thus, power consumption for “on” and “ready” states and the corresponding thresholds stated in Table 15 and Table 16 confirm specific optical network device conformity to EE standards. Also, Table 15 and Table 16 present EE per bit for analyzed ONU/gateway (router) or OLT devices, respectively, which are determined by dividing the “on” state power consumption by the device’s nominal data transmission capacity [56]. The instantaneous power consumption and calculated energy-per-bit levels presented in Table 15 and Table 16 represent EE levels that are characteristic of optical access network devices used in the analyses, which were manufactured in 2024. In subsequent sections, those power consumption levels with 2024 EU CCECBE EE thresholds were used for modeling the power consumption of the ONU/gateway (router) and OLT devices.

#### 6.2.1. Modeling the Power Consumption of the ONU/Gateway (Router) Device

On the subscriber side of the optical FTTH network, the ONU functions are performed by the gateway (router) as a standard customer premises equipment (CPE) device that is responsible for terminating the optical connection and routing traffic between the subscriber’s local network and the operator’s access network (Figure 1). Kontron d.o.o. (formerly Iskratel d.o.o.)’s Innbox U92 universal home ONU (gateway) is selected as a CPE device in this analysis (Table 15), since it integrates functions of the ONU device and home gateway (router) [55]. The Innbox U92 ONU device contains direct small form-factor pluggable (SFP) transceiver connections that enable its implementation as an ONU device and gateway/router in GPONs and P-t-P FTTH networks [55]

The power consumption profile of the ONU Innbox U92 universal gateway is presented in Table 15 [55]. The instantaneous power consumption is estimated to be 9.15 W based on the assumption that half of the time, the ONU/gateway (router) device operates in the “on” state, while in the remaining half of the time, it operates in the “ready” state (Table 15). Thus, this power consumption of the ONU/gateway (router) represents the average CPE (ONU site)’s instantaneous power consumption (which includes the power consumption of ONU and gateway (router) functionalities). Therefore, the ONU/gateway device whose power consumption characteristics are presented in Table 15 is used in this analysis as a typical example of the CPE terminals that can be used in both FTTH P-t-P networks and GPONs. The instantaneous power consumption in the “on” state presented in Table 15 refers to the actual measured power consumption of a device operating in a fully operating state. This value is determined through standardized measurements, conducted in accordance with ETSI EN 303 215, as the power consumption at the DC power supply input of the device. In contrast, “maximum allowed power consumption” in Table 15 denotes the upper instantaneous power consumption limit that a device must not exceed during normal operation according to CCECBE v9.0, Tier 2024. According to Table 15, the power consumption of an analyzed ONU/gateway device in “on” and “ready” operating states is fully in compliance with CCECBE v9.0, Tier 2024, specifications.

#### 6.2.2. Modeling the Power Consumption of the OLT Devices

In the case of power consumption analyses of OLT devices, Table 16 presents power consumption profiles of the OLT devices that are selected for analysis. The OLT devices used in these analyses represent a family of advanced OLT devices designed to deliver broadband services in FTTH networks. This includes high data transmission capacity, flexible configuration options, and optimized EC, which are the main OLT device technical characteristics desirable in the realization of modern optical access networks.

**Table 16:** Detailed overview of the characteristics of the OLT devices [56,57].

Device	Type	Instantaneous Power Consumption (W) (On State)	CCECBE Maximum. Allowed Power Consumption (W)	Utilization Percentage of Max. Allowed Power Consumption (%) (On State)	Maximum Capacity/Overall Number of Ports	Energy Per Bit (nJ/bit)
Iskratel (Kontron) Lumia C16	OLT switch	155	198 (On)	78	150 Gbps/16	1.033
Iskratel (Kontron) Lumia T14	Multi-blade OLT	1851	2021.2 (On)	92	3.4 Tbps/208	0.544

Special attention in the selection of OLT devices used for analyses has been given to OLT device EE, which is crucial to reducing operating costs and ensuring optical network sustainability. The EC characteristics of the analyzed OLT devices are presented in Table 16 [56]. According to Table 16, the instantaneous power consumption of all OLT devices used in the analyses complies with CCECBE v9.0, Tier 2024, recommendations. Also, Table 16 shows that OLT devices with higher transmission capacity tend to achieve better EE per transmitted bit, even though their absolute instantaneous power consumption is higher [56]. In subsequent sections, these levels of instantaneous power consumption are used in the analysis of the FTTH optical network’s global energy consumption.

### 6.3. Results of the Analysis for the FTTH Optical Network’s Global Energy Consumption

#### 6.3.1. Projected Global Distribution of Data Rates

**Figure 10:**
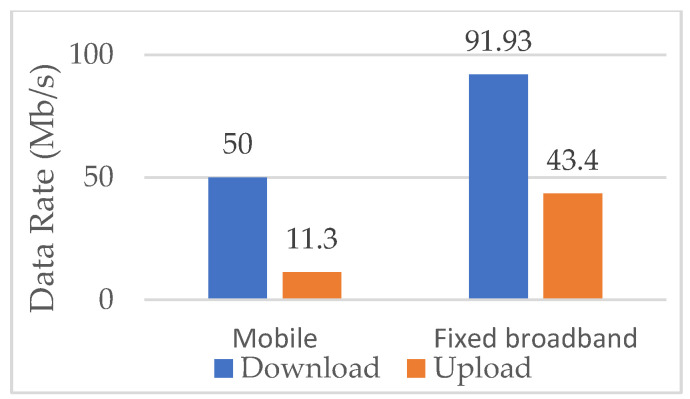
Estimated global average Internet access data rates in 2024 [58].

For the estimation of the global FTTH network EC, the global distribution of FTTH subscribers (connections) has been determined. According to [43], the number of Internet users reached 5.30 billion in 2024, accounting for 66% of the global population. Also, the average data rate in fixed broadband networks is estimated at 91.93 Mbps for download and 43.4 Mbps for upload [58]. Figure 10 illustrates the average data rate for both mobile and fixed broadband services. In this context, fixed broadband refers to high-speed wired technologies such as fiber-optic, digital subscriber line (DSL), and cable access. According to Figure 10 [58], the average Internet access data rate per user in wired access networks in 2024 is estimated at 67.67 Mbps.

#### 6.3.2. Projected Global Distribution of FTTH Subscribers (Connections) and Average Data Rates

This subsection analyzes the anticipated growth of FTTH subscriptions from 2024 to 2035, with a focus on the implications of global subscriber distribution and data rate evolution. According to [59], the global population in 2024 was 8.19 billion (with an annual growth of 0.9%), while [60] reports that 66% of this population was active Internet subscribers. To calculate the future evolution of Internet access speeds in optical networks, the following exponential growth model is applied [61]:

(3) $$R_{avg,t}=R_{avg,2024}\cdot {\left(1+r_{R}\right)}^{t-2024}$$

where $R_{avg,t}$ is the average fixed broadband Internet data rate per subscriber in year t, and $R_{avg,2024}=67.67\;Mbps(baseline)$ is the average Internet data rate per subscriber in 2024. The compound annual growth rate $r_{R}$ is calculated according to

(4) $$r_{R}={\left(\frac{R_{2024}}{R_{2019}}\right)}^{\frac{1}{5}}-1\approx 0.079\;$$

where $R_{2019}=45.0\;Mbps$ is the average Internet data rate per subscriber in 2019 [62]. The year 2019 was selected as the baseline year for calculating the compound annual growth data rate, since this year sets the 7-year referent analysis period (2019–2025), which is statistically relevant in terms of accuracy. Also, 2019 represents the year in which widespread mass-market Gigabit FTTH deployment acceleration occurred worldwide, and this year represents the end of the first generation of GPON dominance and the beginning of the next generation (10 Gbps XGS-PON standardized in ITU-T G.9807.1) of optical access network implementations.

**Figure 11:**
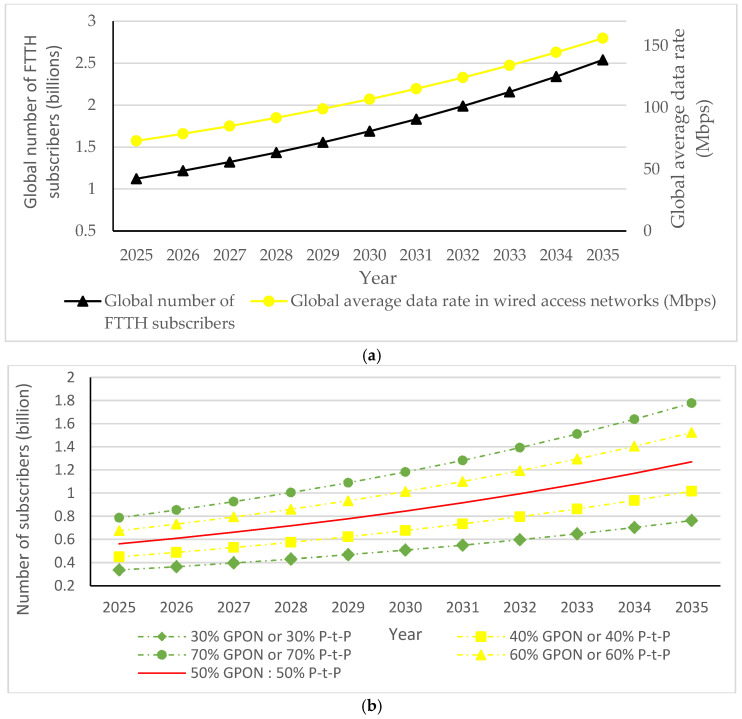
(**a**) Projected evolution of the global number of FTTH subscribers and the global average data rate in optical access networks for the period 2025–2035. (**b**) Projected growth of FTTH P-t-P and GPON subscribers in the period 2025–2035 under different subscriber distribution scenarios.

According to [63], fixed broadband connections were estimated at 1.46 billion in 2024, of which 70.9% were FTTH-based. This corresponds to 1.035 billion global FTTH connections in 2024. The projected annual growth rate for the yearly global increase in FTTH connections is 8.5% [63]. The global number of FTTH connections is modeled using the following expression:

(5) $$N_{user,t}=N_{user,2024}\cdot {\left(1+r\right)}^{t-2024}$$

where $N_{user,t}$ is the global number of FTTH connections in year t, $N_{user,2024}$ is the global number of FTTH connections in 2024 ($N_{user,2024}$), and r is the estimated annual growth rate of the global number of FTTH connections (r=0.085). Figure 11a presents the projected global increase in average data rates in optical FTTH wired access networks alongside the expected number of FTTH subscribers for the period from 2025 to 2035.

Figure 11b presents estimated subscriber growth for five assumed FTTH subscriber distribution scenarios based on FTTH network technology split ratios (P-t-P:GPON) equal to 50% P-t-P:50% GPON, 60% P-t-P:40% GPON, 40% P-t-P:60% GPON, 70% P-t-P:30% GPON, and 30% P-t-P:70% GPON. These scenarios capture the uncertainty in future infrastructure deployments and market-driven trends related to the real practical implementation of active (P-t-P) and passive (GPON) FTTH access networks. The inclusion of different distribution ratios enables flexible modeling of trends in the number of FTTH P-t-P and FTTH GPON connections in accordance with possible future technical and market changes.

All optical network subscriber distribution scenarios presented in Figure 11b reflect exponential FTTH subscriber growth, aligned with the assumed increase in average annual data rates (Figure 11a) that users will experience in FTTH networks. Notably, Figure 11a confirms a concurrent increase in both the number of subscribers and average subscriber access data rates, pointing to an escalating increase in overall FTTH network load on the global level. These projections form the quantitative baseline for the subsequent FTTH network EC modeling, presented in the following subsection, and serve as a foundation for evaluating the long-term EE and scalability of FTTH networks on the global level.

#### 6.3.3. Projections of Global Instantaneous Power Consumption of ONU Devices in FTTH Networks

**Figure 12:**
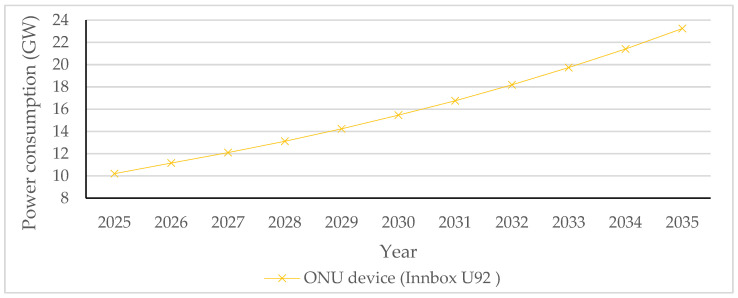
Projected global instantaneous power consumption of ONU devices for the period 2025–2035.

This subsection presents an estimation of the total instantaneous power consumption of ONU/gateway (router) devices at the global level. The analysis incorporates the device-specific ONU power consumption data introduced in Section 6.1 and applies the methodology for calculating power consumption defined in Section 6.2. Considering the fact that at least one ONU device needs to be installed per subscriber, a large number of ONU devices need to be installed worldwide. The number of ONU devices in optical access networks will significantly increase in the future due to the constantly increasing penetration of optical access networks. Figure 12 shows annual projections of the total instantaneous power consumption of ONU/gateway (router) devices (assuming Innbox U92 as ONU device) for the period 2024–2035. The results are expressed in gigawatts (GW) and were calculated by combining the projected subscriber base (50% GPON:50% P-t-P ratio) with the average per-subscriber site power consumption values obtained from Table 15. The total average instantaneous power consumption of ONUs/gateways (routers) on the global level in a specific year is calculated using the following equation:

(6) $$P_{ONU,year}=N_{sub,year}\cdot P_{sub}$$

where $N_{sub,year}$ is the overall number of FTTH subscribers on a global level in a specific year. $P_{sub}$ represents the average estimated instantaneous power consumption per subscriber of the ONU/gateway (router), which includes the power consumption for performing ONU, gateway, and routing functions. The values of $P_{sub}$ are calculated as the arithmetic mean of the device’s “on”-state and “ready”-state power levels, as listed in Table 15. This procedure is repeated annually for each optical device model, using updated subscriber projections and fixed average power consumption values.

Figure 12 depicts the estimated trends in the instantaneous power consumption increase for ONU/gateway (router) devices on a global level. The presented estimations of instantaneous power consumption demonstrate a steady year-over-year increase in the global power demand of ONU/gateway (router) devices at the subscriber side of the network. This trend directly correlates with the exponential growth in FTTH subscriptions presented in Figure 11a.

The results presented in Figure 12 highlight the substantial impact of subscriber growth on access network power demand, reinforcing the importance of energy-efficient ONU/gateway (router) device design and deployment planning in future FTTH network implementations. The results presented in Figure 12 constitute a key input for the comparative evaluation of architectural energy performance in FTTH networks, elaborated on in the next section.

#### 6.3.4. Projected Global Average Number of PoPs and OLT Devices in P-t-P and GPON FTTH Networks

This section evaluates the projected instantaneous power consumption at the global network operator’s PoP level for GPON and P-t-P FTTH network architectures (Figure 1). In this work, the PoP is assumed to be the location of the OLT device(s) with supporting equipment (Figure 1), which includes a rack and power supply for the OLT device. In the analyzed FTTH networks, it is assumed that each subscriber is connected to a single PoP (Figure 1). The methodology used for analyses of the instantaneous power consumption of each PoP containing one or more OLT devices is based on the technical parameters of OLT devices outlined in Table 16 and depends on the subscriber distribution projections presented in Figure 11a,b. In the first part of the analysis, it is assumed for both P-t-P and GPON FFTH networks that each PoP serves a fixed number of subscribers that is equal to 704. Although in practice, single PoPs can serve from 50 to 4000 subscribers per PoP, in this analysis, the value of 704 subscribers served per PoP is assumed to be a typical representative of realistic PoP implementation in practical applications.

##### GPON FTTH Networks

For the GPON FTTH networks, the Iskratel (Kontron) Lumia C16 OLT device (Table 16) from the manufacturer Kontron d.o.o., equipped with 16 user ports, is selected for analysis [56]. This OLT device is selected for analysis since it represents a typical OLT device that will be used in the realization of PoPs of GPON FTTH networks during the upcoming decade. Considering a total OLT device port throughput of 3.75 Gbps (2.5 Gbps downlink and 1.25 Gbps uplink) and a forecasted average access data rate per subscriber equal to 156.18 Mbps in 2035 (Figure 11a), the maximum number of subscribers per port is calculated as a ratio of the maximum port data rate capacity (${DR}_{port})$ and maximum data rate demand per subscriber (${DR}_{sub})\;$at the end of the upcoming decade:

(7) $$N_{sub/port}=\frac{{DR}_{port}}{{DR}_{sub}}$$

It is estimated that a maximum of 23 subscribers can be served by a single port of the Lumnia C16 OLT device.

To align with practical deployment, a 1:32 passive optical splitter per port of the OLT device is assumed to be geographically dispersed in the practical realization of GPON FTTH networks (Figure 1b). In the analyses, the optical splitters are assumed to be located directly at the PoP premises (which is a network structure known in practice as GPON over P-t-P), and such a network architecture results in a centralized splitting configuration (Figure 1b). This analysis considers two Lumia C16 devices per PoP ($N_{OLT/PoP}$), each having 16 ports ($N_{port/OLT})$, which results in the total maximum supported number of subscribers (users) per PoP, expressed as

(8) $$N_{max.sub/PoP}={32\cdot N}_{OLT/PoP}\cdot N_{port/OLT}$$

This maximum number of subscribers per PoP in a configuration with two OLT C16 Lumnia devices (equal to 1024, according to Equation (8)) satisfies the capacity demands of the analyzed PoP with 704 subscribers, which are, for the upcoming decade, equal to

(9) $$N_{sub/PoP}={23\cdot N}_{OLT/PoP}\cdot N_{port/OLT}<N_{max.sub/PoP}$$

where $N_{OLT/PoP}\;=2\;andN_{port/OLT}=16.\;$Therefore, the maximum subscriber capacity of a PoP with two OLT 16-port devices in the analyzed GPON FTTH networks, realized in an architecture with 1:32 passive splitters, exceeds the fixed targeted capacity (of 704 subscribers) that needs to be served per PoP [22] by 2035, allowing space for future access data rate increases and data capacity growth.

##### P-t-P FTTH Networks

In the case of the analyzed P-t-P FTTH networks, a dedicated fiber link for each subscriber is assumed in the P-t-P FTTH network, having an ONU/gateway (router) connection of every subscriber terminated at the OLT located in the PoP (Figure 1a). In the analyzed P-t-P FTTH networks, there are no active or passive network elements that separate or merge optical signals between the OLT located in the PoP and the subscriber ONU/gateway (router). The OLT device Iskratel (Kontron) Lumia T14 from the manufacturer Kontron d.o.o is assumed to be used as an OLT device in PoPs of the analyzed P-t-P FTTH networks (Table 16). This OLT device, having 208 ports of 3.75 Gbps (2.5 Gbps downlink + 1.25 Gbps uplink) capacity and aggregated capacity of 3.4 Tbps, is selected for analysis [56], since it represents a typical OLT device that will be used in the realization of P-t-P FTTH networks in the upcoming decade (Figure 1a). The capacity of each OLT device port is more than sufficient to support the maximum projected average user data rate of 156.18 Mb/s per subscriber by 2035. Since each PoP must support 704 subscribers ($N_{sub/PoP})$, the number of needed OLT Lumnia T14 devices with 208 ports ($N_{port/OLT})$ to satisfy the connection of 704 subscribers per PoP in the P-t-P FTTH network is calculated as

(10) $$N_{OLT}=\frac{N_{sub/PoP}}{N_{port/OLT}}$$

**Figure 13:**
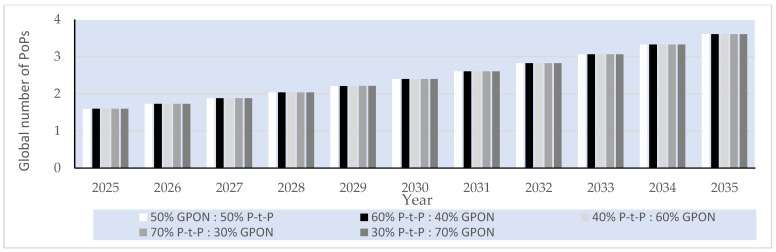
Estimated projections of the global number of PoPs under five FTTH GPON/P-t-P subscriber distribution scenarios over the period 2025–2035.

Based on the calculated number of OLT devices, three Lumnia T14 OLT devices and, additionally, five C16 Lumina OLT devices (Table 16), each having 16 ports, are assumed to be implemented for accommodating a fixed number of subscriber connections (704) and corresponding traffic volume in PoPs of P-t-P FTTH networks up to the year 2035. This configuration guarantees direct connectivity in P-t-P FTTH networks (Figure 13) for all 704 subscribers per PoP, ensuring consistent service quality and stable data transmission across the entire forecast period. The number of PoPs on the global level for the year 2024, for the FTTH network, with a total of 1.035 billion subscribers, was estimated at 1.446.750 PoPs using the following equation:

(11) $$N_{PoP,year}=\frac{N_{sub,year}}{N_{sub/PoP}}$$

where $N_{PoP,year}$ represents the global number of PoPs in a given year, $N_{sub,year}$ is the total number of FTTH subscribers in the analyzed year, and $N_{sub/PoP}$ is the average number of subscribers served by a single PoP (assumed to be fixed and equal to 704).

Figure 13 presents estimated projections of the global number of PoPs under five FTTH GPON/P-t-P subscriber distribution scenarios (ratios) over the 2025–2035 period. The estimation presented in Figure 13 demonstrates how different subscriber distribution scenarios in FTTH P-t-P and GPON architectures directly influence the total number of required PoPs during the period from 2025 to 2035 in a specific FTTH network type. Based on the defined number of PoPs presented in Figure 13, the calculation of the average instantaneous power consumption for GPON and P-t-P FTTH network architectures is conducted in subsequent sections.

#### 6.3.5. Projected Global Average Instantaneous Power Consumption per PoP of FTTH P-t-P and GPONs

Assuming the set of different OLT devices *D =* 1, …, *n*, … *N* installed in a PoP, the total instantaneous power consumption of PoPs in GPONs and P-t-P FTTH networks in a given year *t* was determined for the specific subscriber distribution scenario according to the following relation:

(12) $$P_{GPON,t}=\sum_{n=1}^{N}{P_{OLT}}_{n}\;{\cdot N}_{PoP,GPON,t}$$

where ${P_{OLT}}_{n}$ is the estimated average instantaneous power consumption of the *n*-th OLT device installed in the PoP, and ${N_{PoP,}}_{GPON,t}$ is the estimated number of PoPs on the global level required for the GPON in year *t*. For the GPON FTTH networks, the total power consumption per PoP was calculated assuming the use of two Lumia C16 devices, each with a total instantaneous power consumption of $\sum_{n=1}^{N=2}{P_{OLT}}_{n}=$ 310 W.

**Figure 14:**
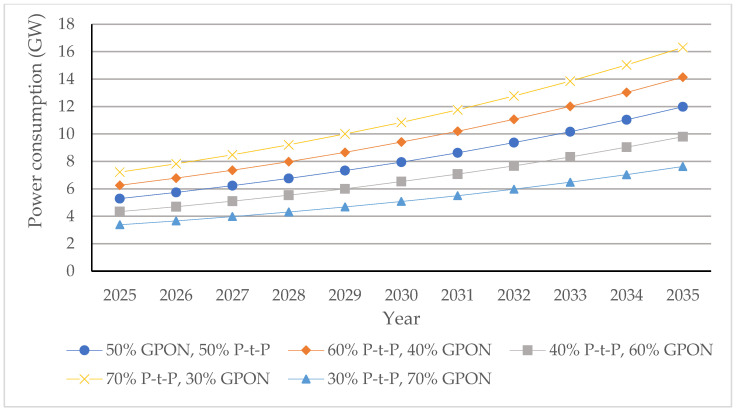
Projected total average instantaneous power consumption of PoPs in FTTH networks under various P-t-P and GPON subscriber distribution scenarios in the period 2025–2035.

For the P-t-P FTTH network, the total power consumption per PoP was calculated by accounting for the use of five Lumia C16 OLT devices and three Lumia T14 OLT devices, with a total nominal power consumption of $\sum_{n=1}^{N=8}{P_{OLT}}_{n}=$ 6328 W. Following the same methodology, the corresponding values were calculated for all subsequent years and subscriber distribution scenarios. The annual instantaneous power consumption of the analyzed FTTH GPON and P-t-P network architectures was projected for the period from 2024 to 2035 under five subscriber distribution scenarios, with the P-t-P-to-GPON subscriber distribution ratio (P-t-P:GPON) in specific scenarios equal to 50%:50%, 60%:40%, 40%:60%, 70%:30%, and 30%:70%. The resulting values, summarized in Figure 14, include yearly estimates for each architecture and scenario of FTTH GPONs and P-t-P networks for each distribution ratio.

Figure 14 shows the increased instantaneous power consumption of PoPs for every analyzed GPON and P-t-P FTTH network subscriber distribution scenario on a global scale. FTTH network architectures having a higher estimated percentage share of FTTH P-t-P networks (60% P-t-P:40% GPON and 70% P-t-P:30% GPON) have significantly higher instantaneous power consumption due to the larger number of network OLT devices with corresponding optical ports that need to be installed in PoPs.

#### 6.3.6. Projected Global Average Instantaneous Power and EC of FTTH P-t-P and GPONs

To assess the global average instantaneous power consumption of the FTTH access network, the global average instantaneous power consumption at the customer premises side (CPE) containing the ONU gateway/router and the global average power consumption of PoPs have been considered. The total global average instantaneous power consumption of the FTTH access network is calculated as the sum of the estimated total instantaneous global ONU gateway/router power consumption and the estimated total global PoPs instantaneous power consumption.

**Figure 15:**
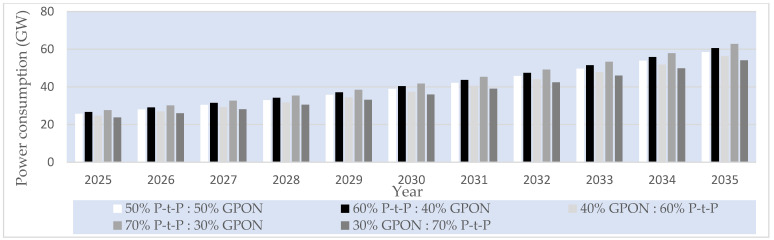
Average instantaneous power consumption of the global FTTH access network for different subscriber distribution ratios of FTTH (P-t-P and GPON) technologies in the period 2025–2035.

Figure 15 provides an overview of the global average instantaneous power consumption trends for various FTTH deployment scenarios in the period 2024–2035, covering different subscriber distribution ratios between P-t-P and GPON FTTH networks. The data presented in Figure 15 shows a continuous increase in total average instantaneous power consumption at the global level over the analyzed decade, with variations depending on the subscriber distribution ratio among P-t-P and GPON types of FTTH networks. In particular, the FTTH P-t-P network requires a larger number of OLT devices per PoP due to the network architecture that is based on a direct connection between the port of the OLT device and each ONU at the subscriber side. This contributes to the higher average instantaneous power consumption on the global level of FTTH network types having a higher distribution of P-t-P FTTH subscribers for every year during the analyzed decade (Figure 15).

**Figure 16:**
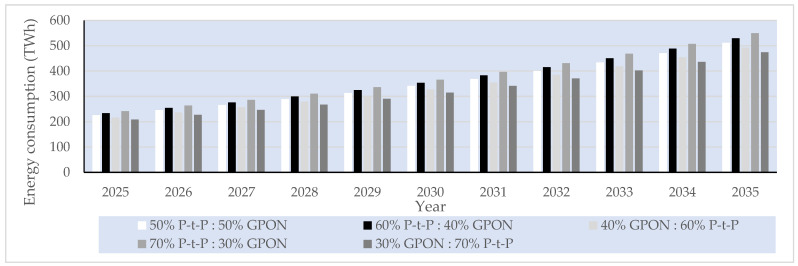
Graphical representation of the total average yearly EC of the global FTTH access network for different subscriber distribution ratios of FTTH (P-t-P and GPON) technologies in the period 2025–2035.

In addition, analyses of annual global FTTH network (P-t-P and GPON) EC are presented in Figure 16. According to Figure 16, the global EC of optical access FTTH networks will continually increase by 2035 for both types (GPON and P-t-P AON) of analyzed FTTH networks, independent of the implementation of methods and techniques that will contribute to reducing the instantaneous power consumption of individual ONU or OLT devices. This is a consequence of the vast increase in the number of FTTH subscribers in the analyzed period.

In accordance with the results obtained for annual instantaneous global FTTH network power consumption (Figure 16), the analyzed network types with a large share of the P-t-P FTTH network type (70% P-t-P:30% GPON, 60% P-t-P:40% GPON) have higher annual EC. This is a consequence of the greater infrastructure and power demands of P-t-P FTTH network architecture. Conversely, Figure 16 shows that the FTTH network configurations having a larger GPON share in the distribution of subscribers (70% GPON:30% P-t-P, 60% GPON:40% P-t-P) yield lower annual EC levels, confirming that the GPON type of FTTH networks is more energy-efficient than the P-t-P type of FTTH networks.

This outcome reflects the benefits of greater subscriber aggregation, a lower number of OLT devices and corresponding optical ports, and lower instantaneous power requirements characteristic of passive (GPON) FTTH network deployments. The results also highlight the significant impact of architecture choice (FTTH GPON vs. FTTH P-t-P) on overall FTTH network energy demand and, consequently, network EE. These estimation results outline how EC in FTTH networks is influenced by network type and scaled by subscriber growth and thus provide key insights for EE network planning.

### 6.4. EC Analysis of FTTH Networks for Different PoP Capacities

While the previous section analyzes EC of the FTTH network under the assumption of a uniform PoP configuration with a fixed capacity related to serving 704 subscribers, real-world PoP deployments vary in the number of served subscribers. Thus, in this section, the analysis is further refined by categorizing the impact of PoP subscriber capacity on FTTH network EE. This enables a more detailed assessment of FTTH network EE across different POP subscriber distribution scenarios. Therefore, this section extends the analysis by incorporating multiple PoP classes differing in their capacity to serve different numbers of subscribers. The classification includes six commonly observed PoP classes in terms of the number of served subscribers, which can be equal to 50, 100, 250, 500, 750, and 1000 subscribers served per single PoP. Taking into account PoPs with different capacities enables a more realistic quantification of how PoP size affects the annual EC.

#### 6.4.1. Impact of PoP Capacity on Instantaneous Power Consumption in FTTH Networks

Based on PoP class capacity needs, a different number of OLT devices (presented in Table 16) were selected to satisfy a specific number of subscribers and corresponding capacity demands for each PoP class. The number of OLT devices in FTTH GPONs was calculated based on relation (10), which states that the maximum number of served subscribers per OLT port with 3.5 Gbit/s capacity is 24 subscribers. Using this constraint, which can be satisfied with the connection of ONU devices over 1:32 passive splitters per each port of the OLT device, the number and type of OLT devices used for the realization of a PoP serving a specific number of subscribers are presented in Table 17.

Additionally, the number of OLT devices in the FTTH P-T-P network is calculated based on the one-OLT-to-one-ONU optical network port connection principle. Thus, the number of OLT optical network ports in the analyzed FTTH P-t-P network needs to accommodate the connection of at least one ONU subscriber, the number of OLT devices, and the corresponding instantaneous power consumption in the active (“on”) operating state per POP.

The analyses assume that the total number of FTTH subscribers in 2025, estimated at 1.123 billion, is evenly distributed across six PoP classes, resulting in 1.87 million subscribers per PoP class. Within each class, a 50% GPON:50% P-t-P distribution of FTTH subscribers is assumed, allowing for balanced energy performance comparisons. The number of PoPs in 2025 and 2035 for each of the six PoP classes was calculated using Equation (11). Based on this calculation and the equal distribution of subscribers (50% GPON:50% P-t-P), Table 18 presents the estimated global number of FTTH PoPs per POP class and served subscribers in 2025 and 2035, respectively. According to the estimated number of PoPs (presented in Table 18) and the instantaneous power consumption of each POP class (presented in Table 17), the global average instantaneous power consumption for each PoP class was calculated using Equation (12).

**Figure 17:**
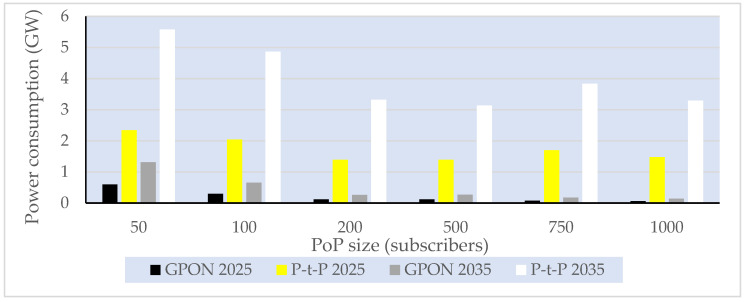
Estimated total global average FTTH access network (ONU + PoP) instantaneous power consumption per PoP size in the period 2025–2035 with a 50% GPON:50% P-t-P subscriber distribution ratio.

To estimate the total average instantaneous power consumption in the FTTH access network for the years 2025 and 2035 per PoP capacity class, a calculation was conducted by summing the power consumption of both the OLT equipment at the PoP and the ONU located at the subscriber premises. For each PoP class, the total access network power consumption was calculated separately for FTTH GPON and P-t-P architectures as the sum of the ONU-side consumption (based on the number of subscribers) and the PoP-side consumption (based on the number and type of OLT devices). The ONU power consumption was calculated using the Innbox U92 ONU device, which supports both GPON and P-t-P modes and has an average power consumption of 9.15 W (Table 15). The ONU-side consumption for each PoP class was obtained by multiplying the number of subscribers (in millions) by 9.15 W per subscriber. Figure 17 illustrates the impact of FTTH network architecture, comparing GPON and P-t-P configurations, on the total global instantaneous power consumption across different PoP capacity classes. The results confirm that P-to-P deployments consistently require significantly higher power compared to GPONs, particularly for PoPs with a smaller number of subscribers, as they lack shared transmission infrastructure and rely on dedicated active components for each user.

By accounting for both ONU and PoP power consumption, Figure 17 provides a comparative overview of total access network energy demand for each PoP size under a balanced subscriber distribution scenario. The trend highlights the EE advantage of GPONs, especially in deployments with higher and medium subscriber densities, where shared infrastructure enables more efficient use of network resources.

**Figure 18:**
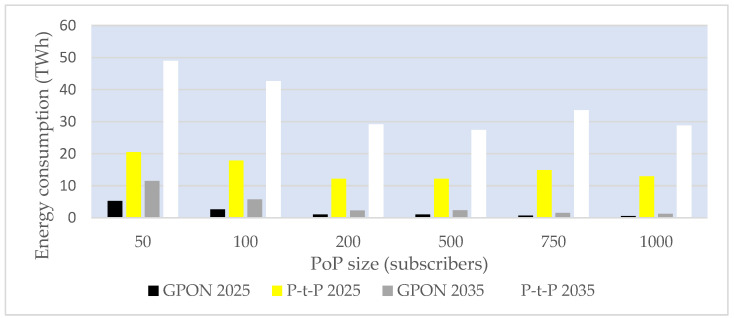
Total global annual EC of the FTTH access network (ONU + PoP) for different PoP classes in the period 2025–2035, with a 50% GPON:50% P-t-P subscriber distribution ratio.

As seen in Figure 17, P-t-P FTTH network architectures exhibit consistently higher instantaneous power consumption compared to GPON architectures. GPON has significantly better EE due to splitter-based user aggregation and the PON elements. Results for the overall annual global energy consumption of EC of P-t-P and GPON FTTH networks per PoP class are presented in Figure 18.

The results indicate that P-t-P-based deployments exhibit significantly higher annual EC than GPON across all PoP size classes in the first and second years of analyses, particularly in low-capacity scenarios (e.g., PoPs with 50 and 100 subscribers). This is a direct consequence of the point-to-point architecture, where each subscriber requires a dedicated optical port and associated hardware for a high number of PoPs with low subscriber capacity. In contrast, GPON architecture demonstrates notably lower energy requirements for each of the analyzed PoP subscriber classes, particularly in scenarios with high and mid-sized PoP capacities, due to the use of optical splitters and shared transmission resources. With increasing PoP capacity (e.g., 750 and 1000 subscribers), the difference in total power consumption between GPON and P-t-P architectures becomes higher, reflecting enhanced equipment utilization and improved efficiency of resource aggregation. These results emphasize the importance of optimal PoP sizing and architecture selection in the design of energy-efficient FTTH networks, with GPONs offering significant advantages in scenarios where large-scale aggregation is feasible.

## 7. Conclusions

This paper presents a comprehensive overview of challenges and solutions for improving EE of optical access networks, with particular focus on FTTH architectures that rely on PONs and AONs. The Introduction emphasized the growing demand for broadband capacity and an increase in the number of FTTH subscribers, which results in a rise in power consumption of optical access network infrastructures. The OLTs and ONUs have been highlighted as the most energy-sensitive elements in optical access networks, and previous activities related to strategies for improving their EE performance have been reviewed.

The paper further reviews the structural and operating characteristics of optical access network technologies and identifies key architectural features and differences between PON and AON deployments. The paper also analyzes standardized ONU energy-saving operating modes, describing techniques such as doze, sleep, cyclic sleep, and watchful sleep, and the impact of adaptive management of sleep mode variants on enhancing optical network EE. These approaches were assessed with respect to their impact on transceiver operating states, traffic-handling capability, operating and wake-up durations, and achievable energy savings. It was shown that proper implementation of these low-power operating states of the ONU device can significantly reduce average ONU power consumption without affecting service quality beyond acceptable limits.

The paper further analyzes advanced resource management techniques for optimizing the energy consumption of ONU and OLT devices. Energy management of OLT/ONU devices based on the standardized watchful sleep operating mode and DBA demonstrated the ability to maintain OLT/ONU responsiveness to network activity while extending the duration of ONU/OLT devices’ low-power operation. The methods for optimizing the OLT EE were also highlighted, as it governs the signaling and synchronization necessary for the proper EC optimization of ONUs. The resource management methods dedicated to optimization of ONU energy consumption, which include reducing the ONU port data rate, decreasing the number of active WLAN antennas, and using the ONU power-shedding method, confirm their potential for ONU power consumption reductions, particularly under bursty traffic conditions. Finally, modeling of the global energy consumption of FTTH GPONs and P-t-P AONs has been presented, comparing their efficiency and scalability at large deployment levels. The modeling was performed for real ONU and OLT network devices and different classes of PoP subscriber capacities. The results show that PON architectures generally achieve lower overall energy consumption due to their passive ODN part, whereas AONs consume more energy because of active elements in the access path. This comparison emphasized that selecting the appropriate PON or AON ODN architecture has significant implications for the sustainability of future broadband access networks.

The work presented in this review confirmed that EE in optical access networks can be substantially improved through a combination of hardware optimization, low-power operating modes of ONU/OLT devices, and intelligent resource management. Future research should extend these findings by exploring the integration of machine learning and predictive algorithms into energy control, as well as by advancing hardware innovations such as photonic integration and energy-proportional transceivers, to further enhance the sustainability of next-generation optical access networks.

## Figures and Tables

**Table 1:** Comparison of the instantaneous power consumption of typical ONU components in PONs [3].

Receiver Component	EPON	GPON	10G-EPON	XG-PON
Avalanche Photodiode—APD (mW)	2.6	2.6	2.6	2.5
Transimpedance amplifier—TIA (mW)	83.4	83.4	123	123
Limiting amplifier—LA (mW)	121	126	145	154
CDR (mW)	545	520	356	356
SERDES (mW)	550	560	~650	~750
Total for receiver (mW)	1302	1292	1500	1800
Back-end circuit (mW)	2700	3150	5850	6750
Entire ONU device (mW)	6000	7000	13,000	15,000

**Table 2:** Comparison of performance and power consumption parameters for GPON, 1G-EPON, 10G-EPON, and XG-PON (10G-PON).

Technology	Standard/Architecture	Max User Rate	Energy Efficiency	Performance
GPON	ITU-T G.984 [27]/P2MP PON	2.5 Gbit/s downstream, 1.25 Gbit/s upstream	~15–25 W/Gbit/s/user; becomes more efficient above ~1500 subscribers due to better resource sharing [6,26].	Moderate performance; suitable for medium-density deployments, limited scaling under high load.
1G-EPON	IEEE 802.3ah [28]/P2MP Ethernet PON	1 Gbit/s symmetric	~20–30 W/Gbit/s/user; more efficient than GPON at low subscriber counts [26].	Comparable to GPON in smaller Ethernet networks; scalability is limited at high traffic levels.
10G-EPON	IEEE 802.3av [29] /P2MP Ethernet PON	10 Gbit/s symmetric	<10 W/Gbit/s/user (for >1000 subscribers); highest efficiency under high-load conditions [26].	Highest EE at high load; high throughput reduces per-user power consumption.
XG-PON (10G-PON)	ITU-T G.987 [30]/P2MP TDM optical network	10 Gbit/s downstream, 2.5 Gbit/s upstream	~15–20 W/Gbit/s/user; energy-efficient due to TDM, DBA, and power-saving modes [26,31].	High performance in moderate to large deployments; slightly less energy-efficient than 10G-EPON but superior to GPON.

**Table 3:** Comparison of performance parameters and power consumption of P-t-P 1 Gbit/s Ethernet, P-t-P 10 Gbit/s Ethernet, and P-t-P 100/200/400 Gbit/s Metro Aggregation Ethernet technologies.

Technology	Standard/Architecture	Max User Rate	Energy Efficiency	Performance
P-t-P 1 Gbit/s Ethernet	IEEE 802.3z [33]; Direct P-t-P Optical Link	1 Gbit/s symmetric	>25 W/Gbit/s per user in small-scale networks; efficiency improves for ≥50 subscribers (users) in MNO central office (CO) and PoP-unconstrained scenarios [26]	Low efficiency in small deployments due to constant switch power consumption; moderate performance for simple access networks
P-t-P 10 Gbit/s Ethernet	IEEE 802.3ae [34]; Direct P-t-P Optical Link	10 Gbit/s symmetric	~10 W/Gbit/s per user (ideal case); >25 W/Gbit/s when uplink is constrained [26]	High data rate performance in large-scale networks; practically limited by MNO CO and PoP uplink capacity
P-t-P 100/200/400 Gbit/s Metro Aggregation	IEEE 802.3bs [35], IEEE 802.3cu [36]; coherent optics with electronic and Digital Subcarrier Multiplexing (DSCM)-based optical aggregation	100–400 Gbit/s	Traditional aggregation: 10.1–12.1 W/Gbit/s; (DSCM)-optimized: ~5.4 W/Gbit/s [37]	Very high throughput; traditional systems exhibit high energy use, while DSCM-based optical aggregation significantly reduces energy consumption

**Table 4:** Total power consumption of the access network path from the OLT to the ONU for PON and AON architectures and the power-ignoring method of operation [39].

Network Type	Ideal Case	Worst Case	Optimized Case
PON	8.34 W	21.99 W	14.61 W
AON	15.21 W	29.16 W	21.81 W

**Table 5:** Total power consumption of the access network path from the OLT to the ONU for PON and AON architectures with the implemented power-saving method of operation [39].

Network Type	Ideal Case	Worst Case	Optimized Case
PON	6.135 W	12.825 W	9.435 W
AON	15.21 W	20.025 W	16.335 W

**Table 6:** Differences in power consumption between PON and AON main network devices (OLT and ONU) in active and sleep modes of operation, and OLT/ONU power consumption per user [40].

Architecture		ONU Power Consumption (Active Mode)	ONU Power Consumption (Sleep Mode)	OLT Power Consumption per User (Active Mode)	OLT Power Consumption per User (Sleep Mode)
AON		3.5 W	3.0 W	0.87 W	0.47 W
PON	WDM PON	4.7 W	3.0 W	1.78 W	0.68 W
	TWDM PON	5.5 W	3.0 W	0.625 W	N/A

**Table 7:** Operating modes of ONU devices and corresponding characteristics.

Method	Description	Characteristics
Cyclic sleep	Transmitters and receivers are periodically turned off, allowing the device to enter a low-power state.	The OLT uses a unicast or broadcast sleep PLOAM message for controlling the transitions among active and sleep periods, and this transition needs to be synchronized with all the ONUs in the cyclic sleep mode.
Sleep/deep sleep	Exploited when network traffic is minimal or absent. Most active ONU device components are turned off, except those following wake-up signals.	Resuming the ONU device from deep sleep requires additional time but provides the greatest energy savings. It is ideal for longer periods of ONU inactivity.
Doze power-saving technique	A lightweight power-saving technique that allows ONU devices to **temporarily disable their optical transmitter** when not in use, while keeping the ONU receiver active to listen for downstream traffic.	Reduces ONU EC while still maintaining connectivity and synchronization with the network.
Watchful sleep	The optical transmitter is turned off, but the optical receiver remains active, allowing the ONU to continuously monitor downstream traffic without transmitting.	Optimizes **EE** while maintaining **fast downstream responsiveness** and eliminates frequent wake-up penalty.

**Table 8:** Comparison of activity states and power savings for different ONU operating modes.

Mode	Optical Transmitter Operating State	Optical Receiver Operating State	Wake-Up Time	Typical Duration of Operating Mode	Operating Mode Power Savings	Operating Mode Traffic Handling Capability
Doze	OFF	ON	Very short (μs–ms)	Short (ms–s)	Low to moderate	Can receive downstream, wakes for upstream
Sleep	OFF	Periodically ON	Medium (ms)	Moderate (s–min)	Moderate to high	Periodic listening for downstream
Cyclic sleep	OFF	OFF (most of the time); ON periodically	Medium (ms)	Moderate (s–min)	High	Periodic wake-up slots to check traffic
Watchful sleep	OFF	ON (but no active transmission)	Short to medium	Moderate	Moderate	Listens for downstream triggers; avoids frequent waking
Deep sleep	OFF	OFF	Long (ms–s)	Long (min–h)	Maximum	No traffic handled until wake-up

**Table 9:** Comparison of IWU and DWU methods in ONU power management.

Characteristics	IWU	DWU
Reaction to the package arrivals	Instantaneous	Delayed for a predefined time
Energy savings	Small	High (up to 80% at low traffic)
Delay	Very low	Moderate, depending on the delay time
Wake-up time of ONU ($H_{ONU})$ and OLT device ($H_{OLT})$	The $H_{ONU}$/$H_{OLT}$ ratio is not a key factor	The $H_{ONU}$/$H_{OLT}$ ratio is a key parameter for optimizing savings and delays
Overall performance	It provides fast responses but less energy savings	Significant energy savings with acceptable latency

**Table 10:** Overview of the energy management phases for ONU and OLT devices in PONs.

Device	Phases	Description
ONU	Active Phase	The ONU is fully operating and processes incoming traffic.
	Power-Saving Phase	The ONU enters a reduced power consumption state.
OLT	Awake Free	The OLT is fully active and operating.
	Low-Power Watch	The OLT monitors network activities with reduced power consumption.
	Awake Forced	The OLT goes into a fully active state to process the resource management requests.
	Alerted Watch	After a delay ($H_{OLT}$), the OLT actively sends FWI wake-up signals to the ONU to trigger activation.

**Table 11:** Advantages and disadvantages of GIANT, HYRA, Modified Max-Min Fair, and static allocation algorithms in OLT and ONU devices [50].

DBA Algorithm	OLT Advantages	OLT Disadvantages	ONU Advantages	ONU Disadvantages
GIANT	Optimal bandwidth allocation for services of different priorities, minimizes latency	Generates more “idle” frames (3.06–3.12 MB per ONU)	Allocates resources based on the current needs of the ONU	Weaker adaptation to inactive states, more “idle” frames
HYRA	Effective reduction in unnecessary resource consumption, the least number of “idle” frames	Slightly higher data transfer latency (0.68–0.9 ms)	Reduces the unnecessary active time of ONU devices, optimizes power consumption	Slightly higher latency in data transfer
Modified max–min fair	Fair allocation of resources among ONUs	Significant transmission delays (up to 109 ms), more “idle” frames	Fair distribution of resources proportional to the data traffic	Poor idle state management, increased energy consumption
Static allocation	Minimal transmission delays due to simple implementation	Generates the largest number of “idle” frames (6.67 MB per ONU)	Easy resource implementation	Unnecessary allocation of resources in inactive states, large energy losses

**Table 12:** Overview of EE methods for OLT devices.

Method	Description	Recommendations and Application
Shutting down inactive elements	Automatic shutdown of inactive ports and modules during low network activity. Enables a reduction in EC without affecting active optical connections.	For achieving efficient energy management, this method is recommended to be applied during periods of reduced traffic (e.g., night periods) or during periods of predictable traffic reductions.
Adjusting the optical split ratio	Redirecting the connection of active ONU devices to a shared port reduces the instantaneous power consumption of the OLT device’s unused ports.	During periods of very low activity, a single OLT port can support many of the ONUs, while the other ports are deactivated.
Changing the port data transfer rates	Dynamic adjustment of the OLT transmission data rate to reduce EC at low network load.	It can be combined with the cyclic sleep technique at the ONU side. It is recommended for additional energy savings. The algorithm monitors the data traffic and adjusts the ONU transmission data rate.

**Table 13:** EE methods for ONU devices.

Method	Description	Application Characteristics
ONU port data rate reduction	Adjusting the port data transmission rate according to the level of traffic load, ranging from higher data rates for larger traffic loads to lower data transfer rates for lower or no traffic (e.g., from 10 Gbit/s to 100 Mbit/s).	This method reduces energy requirements at the physical layer (PHY) of the ONU during periods of lower traffic.
Reducing the number of ONU active antennas	Reducing the active MIMO antenna 4 × 4 configuration up to a 1 × 1 configuration when conditions allow.	Multiple antenna radio frequency (RF) chains transition into an inactive state during low-traffic situations, reducing the power consumption required for multiple RF channels.
ONU power-shedding method	The ONU device reduces power consumption during AC power outages.	The ONU may reduce power consumption by switching off specific ONU interfaces while being powered from a DC power source. Limited contribution to energy savings during loss. AC power supply.

**Table 14:** ONU power and data rate characteristics in different OANs [43].

Technology	Power Consumption (W)	Data Transfer Rate (DS/US)
FE P-t-P	3	0.1 Gbps/0.1 Gbps
1G PtP	3.5	1 Gbps/1 Gbps
EPON	4	1 Gbps/1 Gbps
GPON	5.5	2.5 Gbps/1.25 Gbps
10G EPON	6.2	10 Gbps/1 Gbps
XG-PON	6.5	10 Gbps/2.5 Gbps

**Table 15:** Detailed overview of the characteristics of the ONU/gateway (router) device used in analyses [55].

Device	Instantaneous Power Consumption (On State) (W) (Ready State) (W)	CCECBE Maximum Allowed Power Consumption (W)	Percentage of Max. Allowed Power Consumption (On State) (%) (Ready State) (%)	Maximum Capacity (Gbps)	Energy Per Bit (nJ/bit)	Average ONU Site Power Consumption (W)
Innbox U92	10.8 (On) 7.5 (Ready)	16.5 (On), 11.9 (Ready)	65 (On state) 63 (Ready state)	1	10.8	9.15

**Table 17:** Number of OLT devices per PoP class and corresponding instantaneous power consumption in “on” operating state.

PoP Class (Number of Subscribers)	Number of OLT Devices in FTTH GPON	PoP Average Instantaneous Power Consumption in FTTH GPON (W)	Number of OLT Devices in FTTH P-t-P Network	PoP Average Instantaneous Power Consumption in FTTH P-t-P Network (W)
50	1 × Iskratel (Kontron) Lumia C16	155	4 × Iskratel (Kontron) Lumia C16	620
100	1 × Iskratel (Kontron) Lumia C16	155	7 × Iskratel (Kontron) Lumia C16	1085
200	1 × Iskratel (Kontron) Lumia C16	155	1 × Iskratel (Kontron) Lumia T14	1851
500	2 × Iskratel (Kontron) Lumia T16	310	2 × Iskratel (Kontron) Lumia T14	3702
750	2 × Iskratel (Kontron) Lumia T16	310	3 × Iskratel Lumia (Kontron) T14 + 8 × Iskratel (Kontron) Lumia C16	6793
1000	3 × Iskratel (Kontron) Lumia T16	465	5 × Iskratel (Kontron) Lumia T14	9255

**Table 18:** Number of global FTTH P-t-P and GPON PoPs with an equal number of subscribers (50% GPON:50% P-t-P subscriber ratio) in 2025 and 2035.

PoP Class (Subscribers)	Total Number of PoPs in 2025 (Millions)	GPON/P-t-P Subscribers in 2025 (Millions)	Total Number of PoPs in 2035 (Millions)	GPON/P-t-P Subscribers in 2035 (Millions)
50	3.74	1.87/1.87	8.463	4.232/4.232
100	1.87	0.935/0.935	4.232	2.116/2.116
200	0.93	0.465/0.465	2.116	1.058/1.058
500	0.37	0.185/0.185	0.846	0.423/0.423
750	0.25	0.125/0.125	0.564	0.282/0.282
1000	0.187	0.093/0.093	0.423	0.212/0.212

## Abbreviations

10G-EPON	10-Gigabit Ethernet Passive Optical Network
1G-EPON	1-Gigabit Ethernet Passive Optical Network
AC	Alternating Current
ALA	Advanced Link Aggregation
ALR	Adaptive Link Rate
AON	Active Optical Network
APD	Avalanche Photodiode
AWSM	Adaptive Watch Sleep Mode
BiPON	Bit Interleaving Passive Optical Network
B-CDR	Burst-mode Clock and Data Recovery
B-LA	Burst-mode Limiting Amplifier
B-TIA	Burst-mode Transimpedance Amplifier
BWmap	Bandwidth Map
CCECBE	Code of Conduct on Energy Consumption of Broadband Equipment
CDR	Clock and Data Recovery
CO	Central Office
CoC	Code of Conduct
CPE	Customer Premises Equipment
CPU	Central Processing Unit
CSM	Cyclic Sleep Mode
DBA	Dynamic Bandwidth Allocation
DC	Direct Current
DM	Dynamic Mechanism
DSCM	Digital Subcarrier Multiplexing
DSL	Digital Subscriber Line
DSP	Digital Signal Processor
DWBA	Dynamic Wavelength and Bandwidth Assignment
DWDM	Dense Wavelength Division Multiplexing
DWU	Delayed Wake-Up
EC	Energy Consumption
EE	Energy Efficiency
EER	Energy Efficiency Rating
eOAM	Ethernet Operations, Administration, and Maintenance
EPON	Ethernet Passive Optical Network
ETSI	European Telecommunications Standards Institute
ETSI EN	European Telecommunications Standards Institute—European Norm
FCSM	Fixed Cycle Scheduling Mechanism
FE	Fast Ethernet
FE P-t-P	Fast Ethernet Point-to-Point
FEC	Forward Error Correction
FM	Feedback Message
FTTH	Fiber to the Home
FTTP	Fiber to the Premises
FTTx	Fiber to the x
FWI	Full Watch Interval
GEPON	Gigabit Ethernet Passive Optical Network
GIANT	Grant Independent Allocation with Non-Overlapping Transmission
GPON	Gigabit Passive Optical Network
HSMA	Hybrid Sleep Mode Aware
HYRA	Hybrid Reservation and Allocation
IEEE	Institute of Electrical and Electronics Engineers
ISR	Initiate Sleep Request
ITU-T	International Telecommunication Union—Telecommunication Standardization Sector
IPTV	Internet Protocol Television
IP/WDM	Internet Protocol over Wavelength Division Multiplexing
IWU	Immediate Wake-Up
LA	Limiting Amplifier
LSI	Large Scale Integration
LWI	Link Wakeup Indicator
MIMO	Multiple-Input Multiple-Output
MNO	Mobile Network Operator
MPCP	Multi-Point Control Protocol
MTBF	Mean Time Between Failures
MW-PON	Multi-Wavelength Passive Optical Network
NG-PON2	Next-Generation Passive Optical Network 2
NPC	Normalized Power Consumption
OAN	Optical Access Network
ODN	Optical Distribution Network
OFDMA-PON	Orthogonal Frequency Division Multiple Access Passive Optical Network
OLT	Optical Line Terminal
OMCI	ONU Management and Control Interface Optical Network Coding ONC
ONC	Optical Network Coding
ONU	Optical Network Unit
P2MP	Point-to-Multipoint
PD	Photodetector
P-t-P	Point-to-Point
PHY	Physical Layer
PHY Sync	Physical Layer Synchronization
PIC	Photonic Integrated Circuit
PLOAM	Physical Layer Operations, Administration, and Maintenance
PMD	Physical Media Dependent
PON	Passive Optical Network
PoP	Points of Presence
QoS	Quality of Service
SA	Signal Activation
SERDES	Serializer/Deserializer
SIEPON	Service Interoperability in Ethernet Passive Optical Networks
SMA	Sleep Mode Aware
SNI	Service Node Interface
SPW	Sleep Mode with Periodic Wake-up
SR	Signaling Request
TDM	Time Division Multiplexing
TDM-PON	Time Division Multiplexing Passive Optical Network
TH	Threshold High
TIA	Transimpedance Amplifier
TL	Threshold Low
TLV	Type–Length–Value
Tx	Transmit
TRx	Transmit/Receive
TWDM-PON	Time and Wavelength Division Multiplexing Passive Optical Network
VoIP	Voice over Internet Protocol
WDM	Wavelength Division Multiplexing
Wi-Fi	Wireless Fidelity
WLAN	Wireless Local Area Network
WDM-PON	Wavelength Division Multiplexing Passive Optical Network
WSM	Watch Sleep Mode
WSleep	Watchful Sleep
XOR	Exclusive OR (logical operation)
XG-PON	10-Gigabit-capable Passive Optical Network
XGS-PON	10-Gigabit-capable Symmetrical Passive Optical Network
